# Sterolight as imaging tool to study sterol uptake, trafficking and efflux in living cells

**DOI:** 10.1038/s41598-022-10134-x

**Published:** 2022-04-15

**Authors:** Jarmila Králová, Martin Popr, Jan Valečka, Petr Bartůněk

**Affiliations:** 1 CZ-OPENSCREEN, Institute of Molecular Genetics of the Czech Academy of Sciences, v.v.i., Vídeňská 1083, 142 20 Prague 4, Czech Republic; 2 Light Microscopy Core Facility, Institute of Molecular Genetics of the Czech Academy of Sciences, v.v.i., Vídeňská 1083, 142 20 Prague 4, Czech Republic

**Keywords:** Biochemistry, Cell biology

## Abstract

Information about cholesterol subcellular localization and transport pathways inside cells is essential for understanding and treatment of cholesterol-related diseases. However, there is a lack of reliable tools to monitor it. This work follows the fate of Sterolight, a BODIPY-labelled sterol, within the cell and demonstrates it as a suitable probe for visualization of sterol/lipid trafficking. Sterolight enters cells through an energy-independent process and knockdown experiments suggest caveolin-1 as its potential cellular carrier. Intracellular transport of Sterolight is a rapid process, and transfer from ER and mitochondria to lysosomes and later to lipid droplets requires the participation of active microtubules, as it can be inhibited by the microtubule disruptor nocodazole. Excess of the probe is actively exported from cells, in addition to being stored in lipid droplets, to re-establish the sterol balance. Efflux occurs through a mechanism requiring energy and may be selectively poisoned with verapamil or blocked in cells with mutated cholesterol transporter NPC1. Sterolight is efficiently transferred within and between different cell populations, making it suitable for monitoring numerous aspects of sterol biology, including the live tracking and visualization of intracellular and intercellular transport.

## Introduction

Cholesterol is one of the most important lipid molecules, playing a fundamental structural and functional role in membranes, particularly in the plasma membrane (PM). Its physiological role is indisputable as its dysregulation leads to a wide range of human diseases^1–4^. Cholesterol affects membrane permeability, lateral lipid organization, membrane trafficking and signal transduction; it modulates the activity of various membrane proteins and is the precursor for steroid hormones and bile acids^5–7^. Cholesterol is non-randomly distributed in cells and membranes and it is assumed to participate in the formation of discrete membrane structures such as lipid rafts^8^ and caveolae^9^. The membrane rafts compartmentalize cellular processes by forming platforms through protein–protein and protein-lipid interactions^10^. Lipid raft formation is not limited only to the PM, but lipid raft-like microdomains also occur in ER and mitochondria, where they participates in intracellular lipid and protein trafficking from the ER, Golgi, and endosomes to the PM and facilitate interaction between the ER and mitochondrial membranes for the exchange of lipid and proteins^11^.

Cholesterol is transported in plasma as a component of lipoproteins, especially low-density lipoprotein (LDL), in the limiting phospholipid monolayer, or in an esterified form as a component of the lipoprotein core^7^. The particles are endocytosed into cells by clathrin-coated vesicles via the LDL receptor^12,13^ and transported to acidic endocytic compartments where cholesteryl esters are hydrolysed by acid lipase to provide unesterified cholesterol for cellular needs^14,15^. Intracellular cholesterol transport takes place by vesicular transport requiring energy and by spontaneous diffusion or protein carrier-mediated diffusion^16^, which are non-energy-dependent^17^. Recently, the importance of non-vesicular routes of transfer^18–20^, which are conveyed by soluble carriers called sterol transfer proteins (STPs)^21,22^, and facilitated by membrane contact sites (MCSs) between distinct organelles, has been highlighted^23^.

Cholesterol homeostasis is essential for the functional integrity of the cell and requires a tight regulation of cholesterol levels inside and outside of the cell. The cholesterol-sensing regulatory circuits communicate any alterations in cholesterol levels in order to control sterol synthesis, uptake, transport and efflux^15,24,25^. Since a large portion of intracellular cholesterol is localised in the endocytic-recycling compartment (ERC), this pool is considered important in maintaining cellular cholesterol homeostasis^16^. Other works highlight the role of so-called “active cholesterol”, a fraction of plasma membrane cholesterol that exceeds the complexing capacity of the polar bilayer lipids and exhibits an elevated chemical activity. Active cholesterol redistributes down its diffusional gradient to the endoplasmic reticulum and mitochondria, where it binds multiple effectors and directs their feedback activity as an upstream regulator of cellular sterol homeostasis^26,27^.

Excess cholesterol is managed either by its storage as cholesteryl esters in cytosolic lipid droplets (LDs)^17^or exported outside cells. Cholesterol efflux can be a passive process involving simple diffusion via the aqueous phase and facilitated diffusion mediated by scavenger receptor class B, type 1 (SR-BI)^28^. The active pathways are mediated by the ATP-binding cassette (ABC) transporters ABCA1 and ABCG1, which are membrane lipid translocases^28,29^.

Most information about cholesterol transport to the cell interior were derived from examining the effects of pharmacological agents. Unfortunately, many of them have ambiguous effects and may inhibit other cholesterol transport pathways^30^. The subcellular fractionation and isolation of labelled cholesterol have also limits due to problems with purity and contamination of individual fractions. Direct visualization of cholesterol using fluorescent cholesterol probes and their further development together with the development of microscopic techniques enhancing their sensitivity show great promise. For that purpose various cholesterol analogues^31–38^, sterol binding toxins^39,40^, and anti-cholesterol antibodies^41^ have been already analysed. However, most of the probes developed so far have not yet displayed all the appropriate characteristics for faithful monitoring of cholesterol transport^42,43^. At present CHIMs (cholesterol-based imidazolium salts) have been indicated as very promising, as they allow a flexible on-demand attachment of any fluorophore of choice or other functional groups for cholesterol visualization in live cells without need of cell fixation^44^. In any case, another suitable probe to confirm and expand the existing knowledge about cholesterol trafficking is still very valuable.

In our previous study, we described a new cationic FP-5 sterol probe connected to the BODIPY fluorophore^45^, which we here rename as Sterolight (Supplementary Fig. S1). This probe displayed desirable fluorescence properties, and very fast uptake and trafficking inside cells. Herein, we tried to understand how the Sterolight probe enters cells and what major mechanisms and pathways drive its trafficking. For this purpose, we used various pharmacological inhibitors and gene knockdowns potentially involved in the flux of the Sterolight probe and discussed our results in the context of other published sterol probes.

## Results

### Effect of endocytic pathway inhibitors on Sterolight uptake

**Figure 1:**
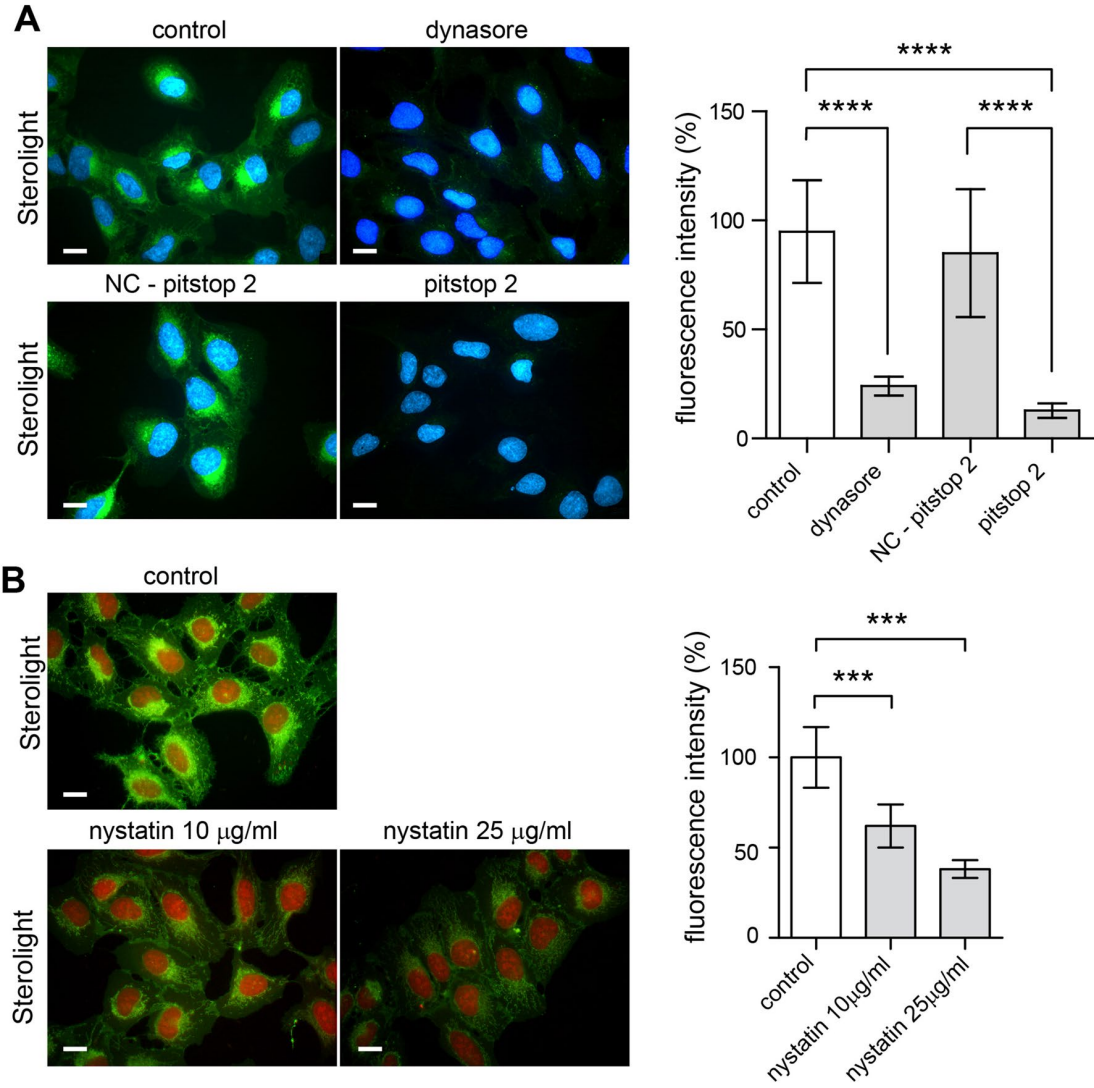
Pitstop 2, dynasore and nystatin inhibit Sterolight uptake. U-2 OS cells were pre-incubated for 15 min in the presence of 10 µM dynasore, 10 µM negative control for pitstop 2 (NC- pitstop 2) and pitstop 2 and (**A**) or nystatin (10 and 25 µM) (**B**) and then pulsed with Sterolight complexed with MβCD for 1 min. After 30 min incubation in the presence of inhibitors, cell nuclei were stained with Hoechst (blue) or Draq5 (red) for 5 min and fluorescence was recorded. The cells were imaged live, and the mean Sterolight fluorescence intensity (%) in control or inhibitor treated cells was analyzed. The bars represent mean ± SEM, and number of cells is 10–20. ***p < 0.001 between control and inhibitor treated cells. Scale bar 10 µm.

Firstly, we determined whether the most commonly known pathway, clathrin-mediated endocytosis (CME), is involved in the uptake of Sterolight probe. Using 10 µM chlorpromazine (CPZ), an inhibitor of this endocytic pathway^46^, we did not see any marked effect on Sterolight uptake in U-2 OS cells. However, the same concentration markedly reduced the uptake of the Alexa 568-transferrin conjugate (A-Tf), which is known to enter cells through clathrin-mediated endocytosis and served as a positive control (Supplementary Fig. S2A, B). Because higher CPZ concentrations in line with previous observations^47^ were toxic to U-2 OS cells, we decided to use other inhibitors such as pitstop and dynasore. Pitstop 2 used to be declared as an inhibitor of clathrin^30^ and dynasore as an inhibitor of dynamin^48^. Both clathrin and dynamin are essential for clathrin-mediated endocytosis. At first, we pre-incubated cells for 15 min with 5–10 µM pitstop 2, negative control to pitstop 2 and 10–80 µM dynasore. Subsequently, cells were pulsed with Sterolight probe and chased for 30 min. A strong Sterolight intracellular signal was detected in control (inhibitor-untreated) cells and negative control-treated cells. In contrast, Sterolight fluorescence of dynasore- and pitstop 2-treated cells was dramatically decreased (Fig. 1A). In addition, incubation with nystatin, which is known as a sterol binding agent disassembling caveolae, also decreased the Sterolight signal (Fig. 1B). This could indicate the involvement of caveolae-mediated Sterolight uptake.

### Knockdown of caveolin-1 and dynamin-2 results in suppression of Sterolight uptake

**Figure 2:**
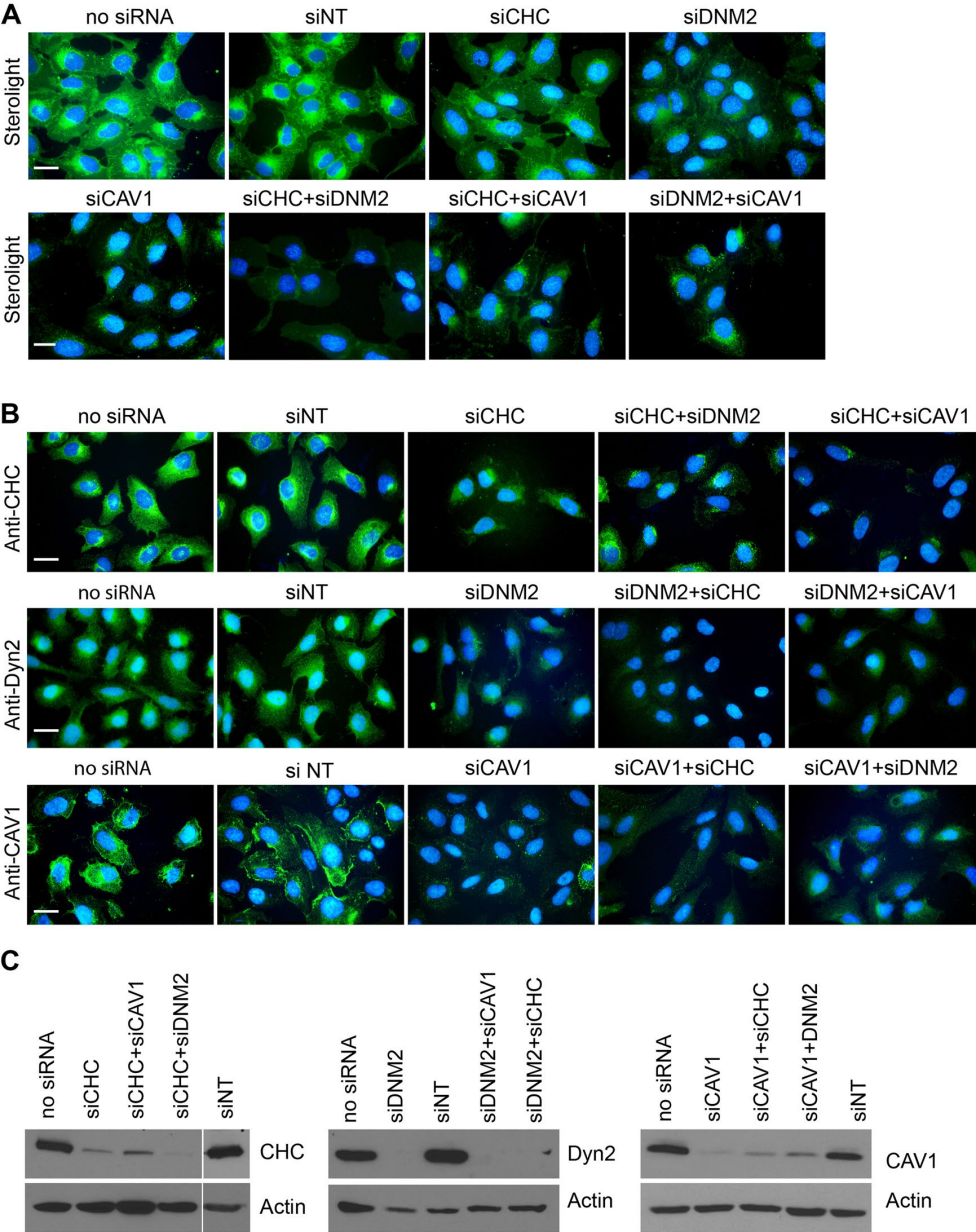
Knockdown of caveolin-1 and dynamin-2, unlike clathrin, leads to suppression of Sterolight uptake. U-2 OS cell were transfected with individual siRNAs targeting clathrin (siCHC), dymamin-2 (siDNM2) and caveolin-1 (siCAV1) or their combinations. (**A**) Transfected cells with silenced expression of specified genes were pulsed with Sterolight probe (1 µg/ml) for 1 min and after 30 min, nuclei of cells were labelled with Hoechst and fluorescence was recorded. For comparison were included cells subjected to transfection procedure without siRNA (no siRNA) or cells transfected with non-targeting siRNA (siNT) as a negative control. (**B**, **C**) The expression of targeted genes was assessed after 96 h by immunostaining with specific antibodies (**B**) or cell lysates were subjected to Western blot analysis (**C**) to confirm that siRNAs effectively reduced protein expression. For spatial reasons, only cropped parts of membranes with bends of corresponding proteins are shown. Full-length membranes with marked edges are presented in Supplementary Information in Fig. S3. The same membranes were subsequently re-probed for actin to verify equal protein loading (lower panel). Cell nuclei were stained with Hoechst for 5 min. Scale bar 10 µm.

We used RNA interference to confirm the mechanism of Sterolight uptake, indicated by our experiments employing small molecule inhibitors. We targeted clathrin heavy chain, dynamin-2 and caveolin-1 using corresponding siRNAs: siCHC, siDNM2 and siCAV1, respectively. Cells were transfected with indicated individual siRNAs or their combinations, while cells subjected to transfection procedure without siRNA (no siRNA) or cells transfected with non-targeting siRNA (siNT) served as negative controls for comparison. Three days later, cells were pulsed with Sterolight probe and their cellular fluorescence was chased after 30 min (Fig. 2A). Cells with depleted clathrin showed similar Sterolight signal as controls, while cells with knockdown of dynamin-2 and caveolin-1 expression exhibited significantly lower uptake of Sterolight. Thus, knockdown of endogenous caveolin-1 led to similar result as nystatin treatment (Figs. 1, 2A). Similar reduction of fluorescent intensity appeared in combinations siCHC + siDNM2, siCHC + siCAV1, and siDNM2 + siCAV1 (Fig. 2A). Simultaneously plated cells from the same transfection were subjected to immunofluorescence using specific antibodies (Fig. 2B) or Western blot analysis using cell lysates (Fig. 2C, Supplementary Fig. S3) to confirm silencing of respective genes. Both methods confirmed substantial, though not complete, decrease of targeted proteins. This experiment shows significant effects of dynamin-2 and caveolin-1 knockdowns on Sterolight uptake and thus support data obtained using the pharmacological inhibitors dynasore and nystatin. On the other hand, clathrin-mediated endocytosis, although clearly demonstrated for transferrin in the same cells (Supplementary Fig. S4), has not been proven for the Sterolight probe. In this case, significant signal reduction was observed only when clathrin was depleted together with caveolin-1 or dynamin-2, but not alone (Fig. 2A).

### Uptake of Sterolight probe is energy-independent

**Figure 3:**
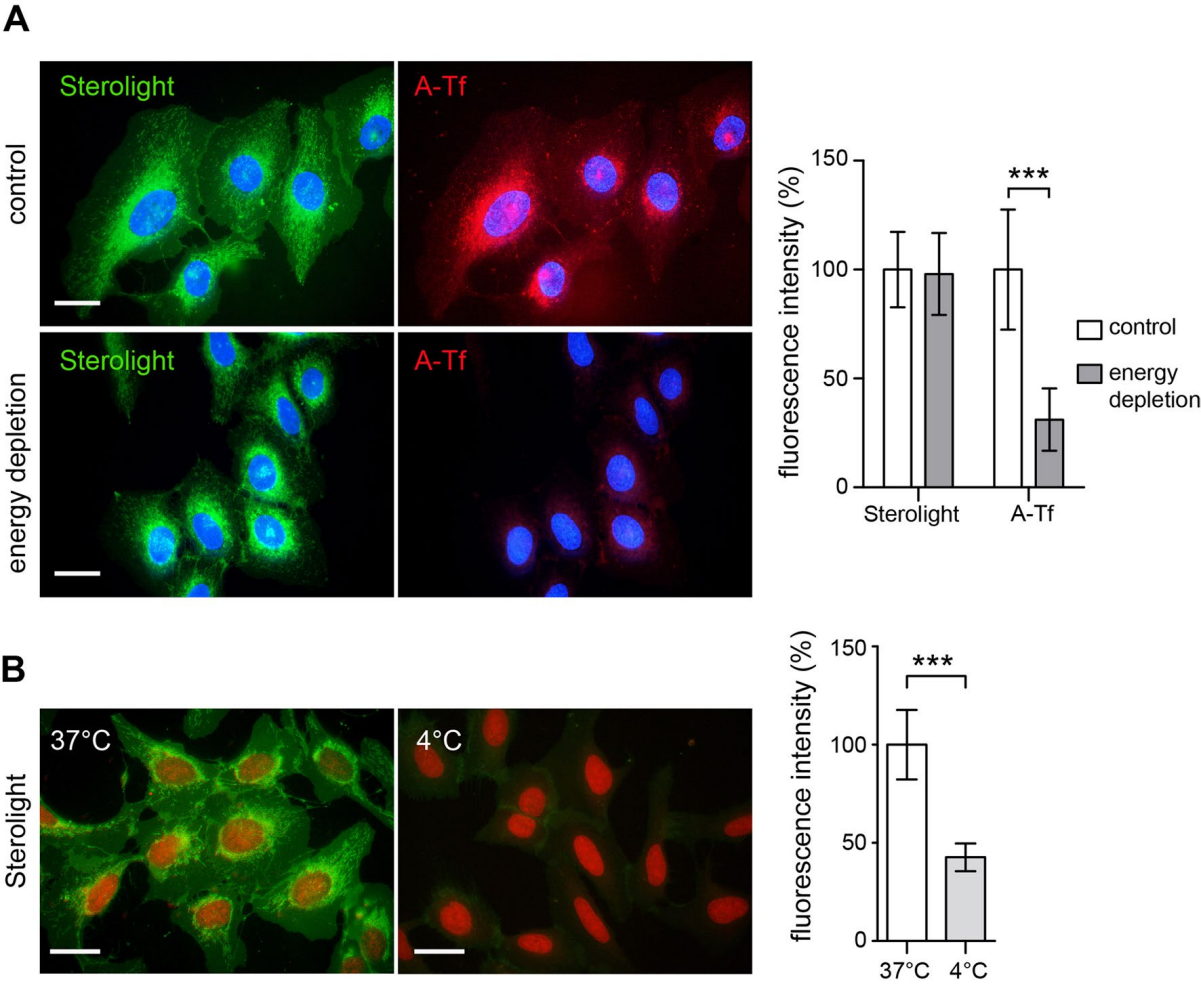
Energy depletion does not lead to suppression of Sterolight uptake. (**A**) U-2 OS cells were energy-depleted by incubation in M2 medium containing energy poisons (50 mM 2-deoxyglucose and 5 mM sodium azide) for 30 min at 37 °C, then pulsed with Sterolight probe (1 µg/ml) for 1 min and chased for additional 30 min in medium containing Alexa 568-transferrin conjugate (**A-Tf**). For last 5 min, Hoechst was added to stain the nuclei. Control cells were incubated in M1 medium containing glucose without energy poisons. The cells were imaged live, and the mean Streolight and Alexa-transferrin fluorescence intensity (%) was evaluated in control or energy-depleted cells. The bars represent mean ± SEM, and number of cells is 10–15. ***p < 0.001 between control and energy-depleted cells. While influx of Alexa 568-transferrin in energy-depleted cells was significantly reduced by energy poisons, Sterolight fluorescence remained unchanged. (**B**) Uptake of Sterolight in the cultivation medium with 5% LPDS was strongly reduced (p < 0.001) at 4 °C. Cell nuclei were stained with Draq5. Scale bar 10 µm.

To determine if Sterolight transport requires energy, we pre-incubated cells in M2 medium containing energy poisons, 2-deoxyglucose and azide, for 30 min. Subsequently, cells were pulsed with Sterolight probe and chased for 30 min in the presence of Alexa 568-transferrin (A-Tf) conjugate. Figure 3A shows that although uptake of A-Tf (5 µg/ml) was in energy-depleted cells substantially reduced, the uptake of Sterolight remained similar as in control cells. It indicates that the cellular influx of Sterolight is mediated by an energy-independent mechanism. Sterolight transport to the plasma membrane and cell interior, however, was stopped at 4 °C (Fig. 3B).

### Sterolight redistribution to lysosomes depends on functional microtubules

In our previous work, we reported very quick and temporary localization of Sterolight probe in some organelles^45^. Here, we validated a significant co-staining of Sterolight with ER probe and some overlaps with mitochondrial probe (Supplementary Fig. S5A, B) early after Sterolight loading. After longer incubation ≥ 1 h, Sterolight signal gradually accumulated in lysosomes as shown by co-localization with LysoTracker Red (Supplementary Fig. S5C) and lastly (within 24–48 h) in lipid droplets (Supplementary Fig. S6). Furthermore, we investigated Sterolight stability over a 1–48 h time period with respect to its location in lipid droplets, detecting both acetylated and converted (hydroxylated) FP-7 form (Supplementary Fig. S7).

**Figure 4:**
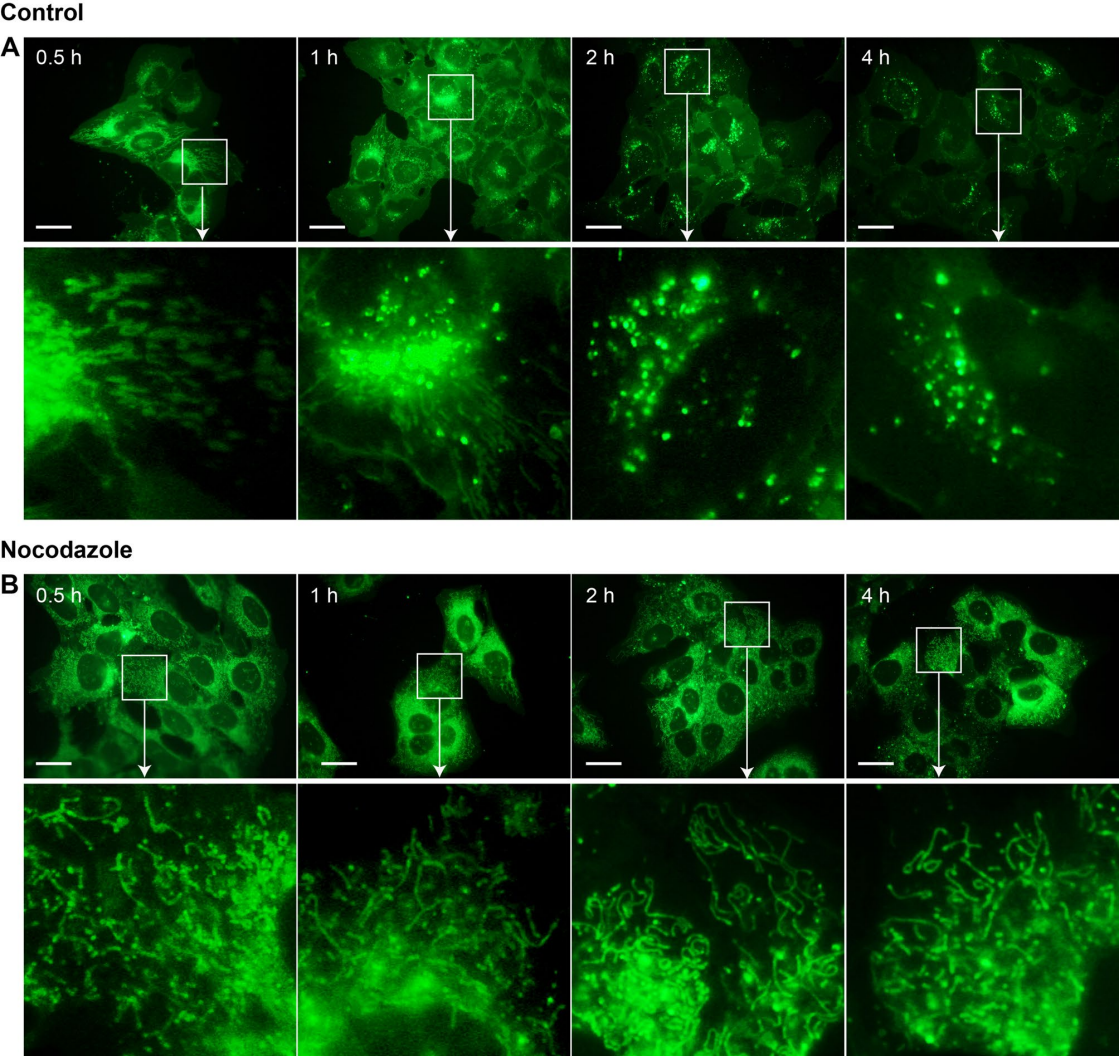
Nocodazole treatment prevents Sterolight redistribution to lysosomes. U-2 OS cells were pulsed with Sterolight probe (1 µg/ml) complexed with methyl-β-cyclodextrin for 1 min, washed, and chased for indicated time at 37 °C in the absence (control A) or presence of 50 µM nocodazole (B). Magnifications of the regions indicated by the white boxes are shown below. The presented results are representatives of three independent experiments. Scale bar 10 µm.

However, when cells were after Sterolight pulse incubated in the presence of microtubule disruptor, 40–50 µM nocodazole, Sterolight signal (Fig. 4B) in contrast to control cells (Fig. 4A) remained in ER and mitochondria even after 4 h incubation and did not notably proceed to lysosomes (Fig. 4B and Supplementary Fig. S8A, B). Thus, for redistribution of Sterolight to lysosomes, functional cytoskeleton is necessary.

### Efflux of Sterolight requires wild type NPC1 cholesterol transporter

**Figure 5:**
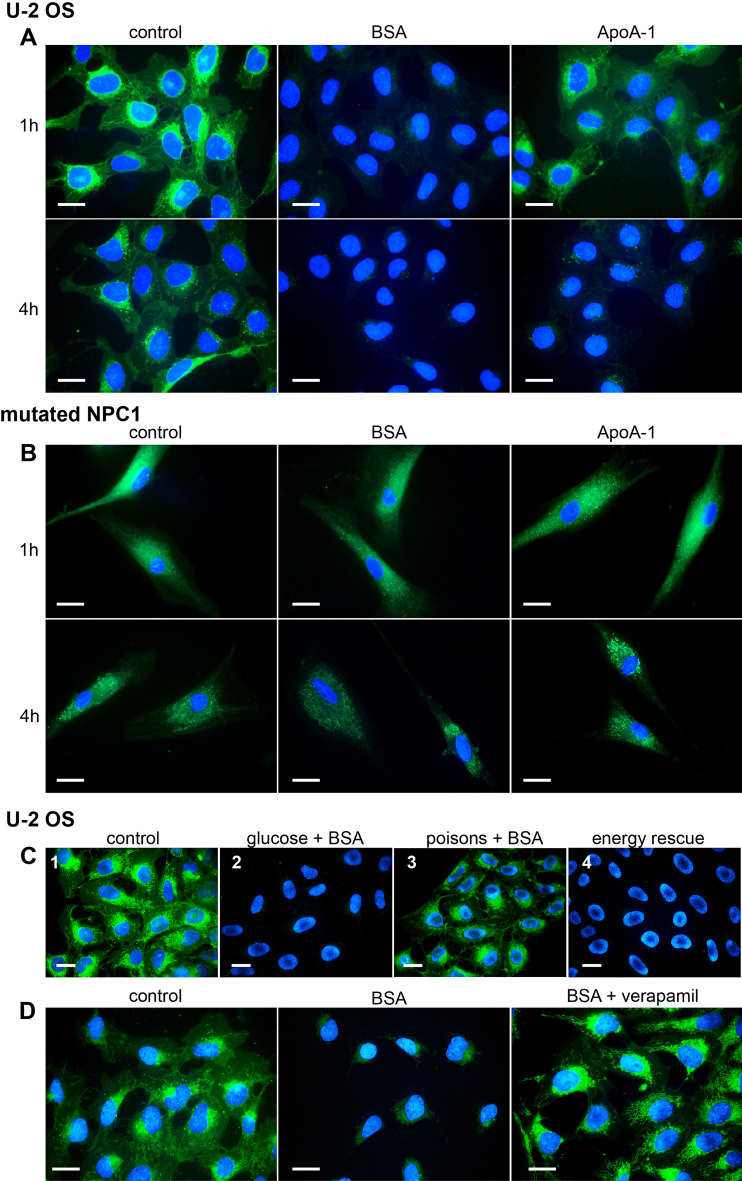
Efflux of Sterolight probe is dependent on energy and functional transporters. U-2 OS cells (**A**) or NPC1 mutated cells (**B**) were pulsed with Sterolight probe (1 µg/ml) and transferred to serum-free medium containing efflux acceptors: 0.2% BSA or 10 µg/mL ApoA-I. The fluorescence intensity of cells was recorded under identical settings after 1 and 4 h incubation. (**C**) Cells pulsed with Sterolight probe were chased for 1 h: 1) in the absence of energy poisons and BSA (negative control), 2) in the presence of BSA (positive control), 3) in the presence of BSA and energy poisons, 4) cells treated with BSA and energy poisons were washed and switched to the medium containing BSA and glucose for additional 1 h incubation to retrieve energy. (**D**) Inhibition of Sterolight efflux by verapamil. U-2 OS cells pre-incubated with 10 µM verapamil for 1 h were pulsed with Sterolight probe, washed and chased in the presence of 0.2% BSA (acceptor) with verapamil for 1 h (right panel). Control cells were labelled with Sterolight probe without BSA or verapamil (left panel). As positive control for efflux were used cells exposed to Sterolight pulse with subsequent incubation in medium containing BSA (middle panel). Cell nuclei were stained for the last 5 min of incubation with Hoechst 33342 (blue). The presented results are representatives of three independent experiments. Scale bar 10 µm.

An important parameter in studying sterol trafficking and homeostasis is the ability of the probe to undergo exchange with extracellular acceptors. To address the release of Sterolight from cells, U-2 OS cells were labelled with probe, then incubated in the absence (control) or presence of acceptors (apolipoprotein A-I, albumin) in serum free medium (Fig. 5A). The Sterolight fluorescence in the cells was evaluated after one and four hours of incubation. Under these conditions, a dramatic reduction of cellular fluorescence was visible already after 1 h of incubation using BSA mediator, indicating high level of Sterolight efflux. With the specific acceptor ApoA-I, which represents the major component of high-density lipoprotein (HDL), a significant reduction of intracellular fluorescence of Sterolight was observed after 4 h incubation (Fig. 5A, bottom row). In cells with mutated cholesterol transporter NPC1 tested under the same conditions, Sterolight efflux was not significant (Fig. 5B).

### Efflux of Sterolight probe is energy- and ABC transporters-dependent

We further analysed whether Sterolight efflux requires energy (Fig. 5C). U-2 OS cells were pulsed with Sterolight probe and chased for 1 h in the presence of BSA in medium M1 containing glucose (Fig. 5C-2) or in medium M2, containing energy poisons, 2-deoxyglucose and sodium azide (Fig. 5C-3). Energy depletion resulted in the inhibition of Sterolight efflux in the presence of BSA (Fig. 5C-3) in contrast to control cells supplemented with glucose (Fig. 5C-2). To show that the block in efflux was specifically due to energy depletion, the energy poisons were washed off and cells were then chased in normal medium containing glucose for an additional 1 h. Under these conditions, efflux of Sterolight probe was restored (Fig. 5C-4). In addition, when cells were treated with the ABC transporter inhibitor-verapamil, Sterolight efflux was suppressed (Fig. 5D, right panel). In this respect, Sterolight efflux is similar to cholesterol, as it requires energy and functional ABC transporters.

### Sterolight transfers between identical and genetically distinct cells

**Figure 6:**
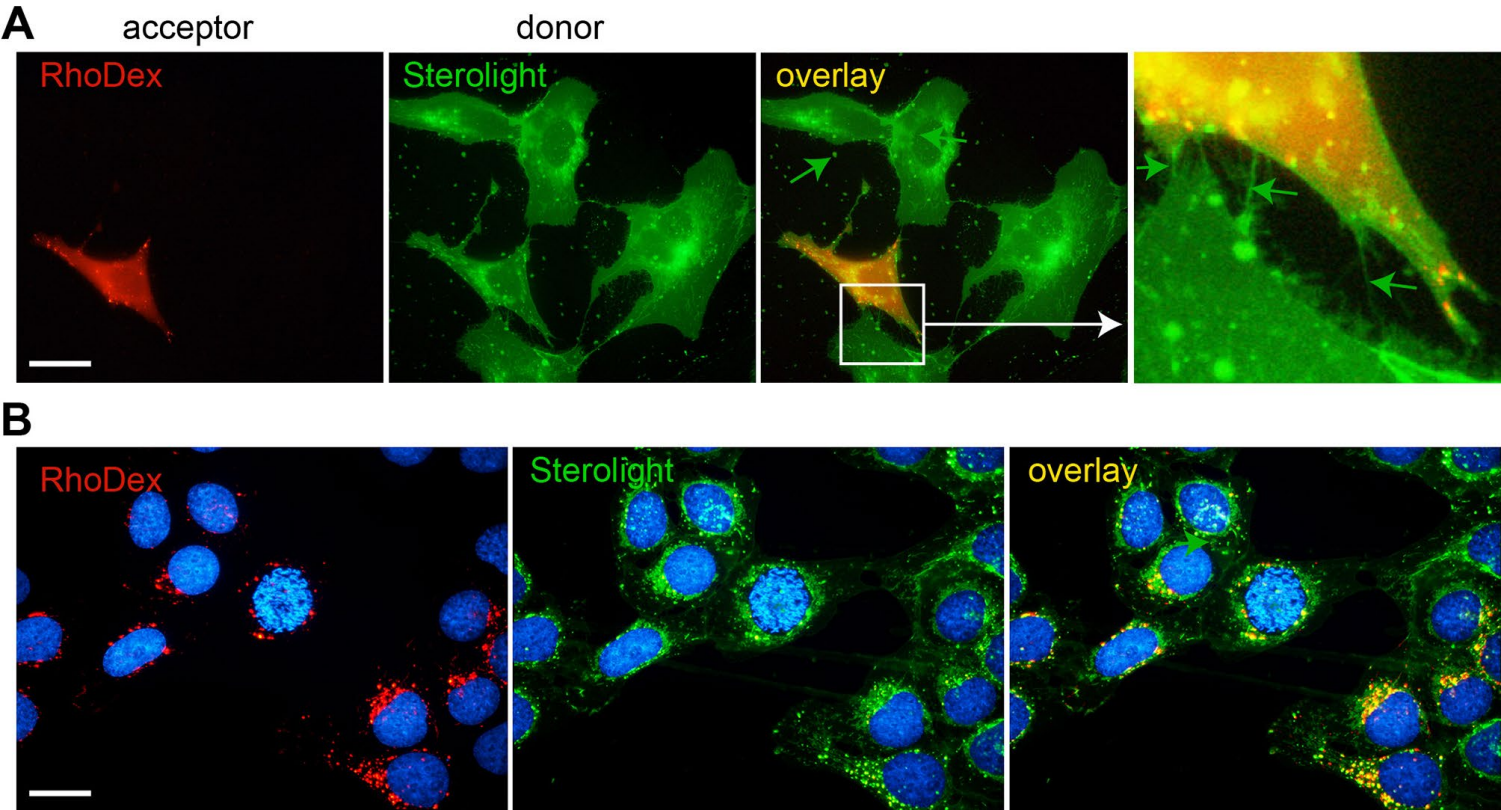
Sterolight transfer from donor to acceptor cells within one cell line. U-2 OS cells labelled overnight with rhodamine-dextran (RhoDex) (1 mg/ml) were harvested and plated on a dish with cells labelled with Sterolight probe via pulse. RhoDex and Sterolight labelled cells were further co-cultivated in FluoroBrite medium supplemented with 5% LPDS for 1 h (**A**) or 24 h (**B**). The fluorescence was recorded under identical settings. Sterolight probe was quickly effluxed from donor cells (green) and taken by RhoDex (red) acceptor cells. Numerous vesicles and nanotubes (indicated by green arrows) formed between donor (Sterolight-labelled) and acceptor (RhoDex-labelled) cells and green vesicles shed from donor cells are visible on enlargement in the right panel. The presented results are representatives of three independent experiments. Scale bar 10 µm.

To study the distribution of the probe between different populations of living cells, we co-cultured U-2 cells, which were separately labelled with rhodamine-dextran (RhoDex) or Sterolight. Onto dishes with Sterolight labelled cells (donor cells—green) were plated cells labelled with rhodamine-dextran (1 mg/ml) (acceptor cells -red). Following attachment (within 30–60 min), Sterolight green label was very effectively sequestrated form donor cells to acceptor cells (Fig. 6A) and remained there 24 h (Fig. 6B) as the probe was released from one cell type and taken by another. Numerous extracellular vesicles shed from donor cells, and tubular structures formed between donor–acceptor cells pointed by green arrowheads, were observed (Fig. 6A, right panel). The same pattern was detected when genetically distinct cells, human U-2 OS osteosarcoma and chicken DF-1 fibroblasts stably expressing fluorescent protein mCherry, were co-cultivated (Supplementary Fig. S9). Both these sets allowed the visualization of sterol transfer between different living cells (Fig. 6A, Fig. S9).

### Sterolight transfer from cells with mutated NPC1 is reduced

**Figure 7:**
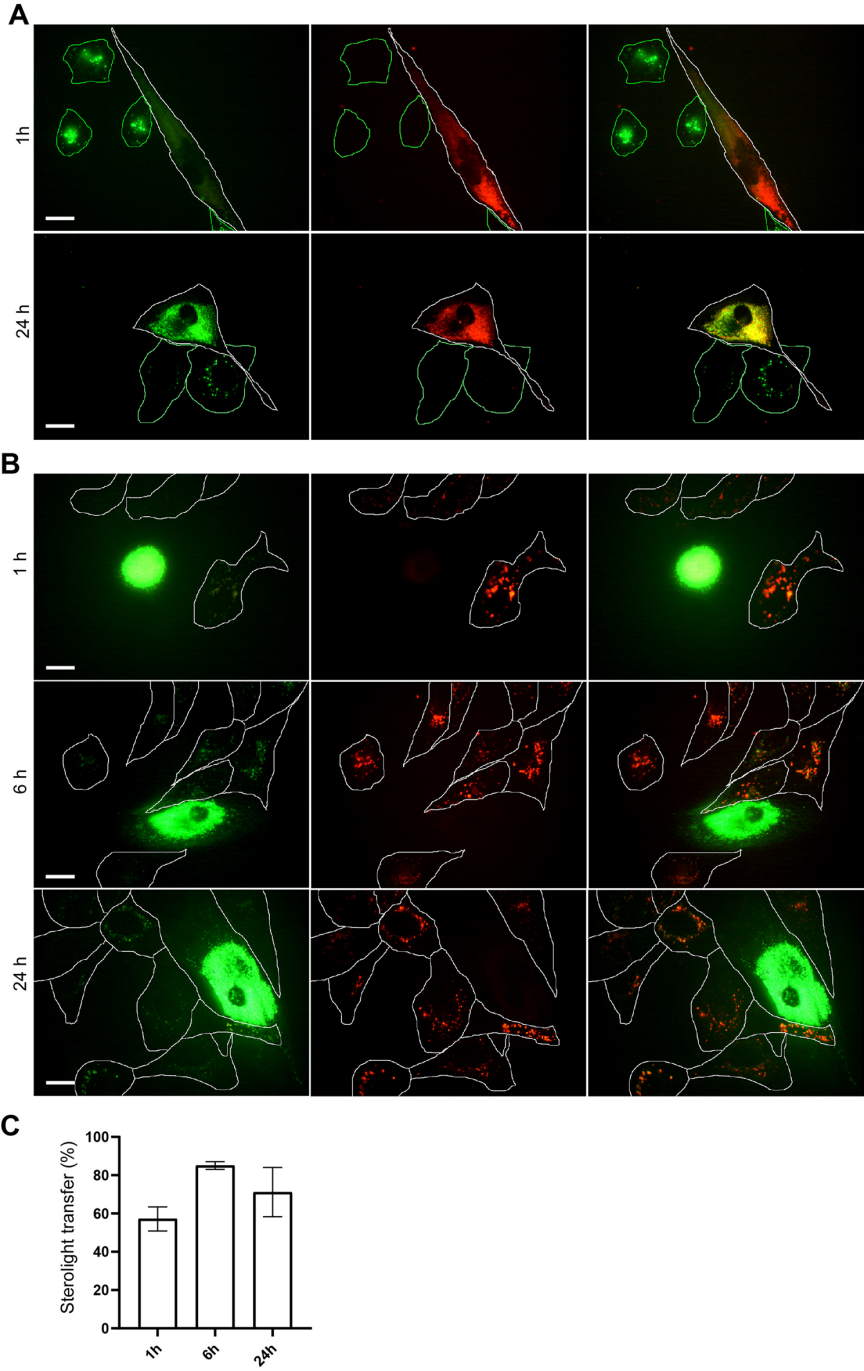
Sterolight transfer between U-2 OS and NPC1 mutated cells. NPC1 cells (clone GM03123) were either labelled with RhoDex (**A**) or Sterolight probe via pulse (**B**) and co-cultivated with inversely labelled U-2 OS cells for 1 or 24 h. (**A**) Sterolight probe originating from U-2 OS cells (outlined green) was detected in 100% RhoDex-labelled NPC1cells (outlined white) within 1 h (**A**, top row). After 24 h, Sterolight signal accumulated mainly in NPC1 cells (**A**, bottom row). (**B**) NPC1 cells labelled with Sterolight probe were, after harvest, plated to RhoDex-labelled U-2 OS cells (outlined) and co-cultivated for 1, 6, and 24 h. Transfer of Sterolight form NPC1 cells to U-2 OS was detectable, but less pronounced (lower fluorescence was evident in 56–85 % of acceptor cells, depending on incubation time, panel **C**). Scale bar 10 µm.

To compare sterol transfer between NPC1 mutated cells and normal U-2 OS cells, cells were separately labelled either with rhodamine-dextran or with Sterolight and co-cultivated with inversely labelled counterpart for 1 and 24 h (Fig. 7). NPC1 cells labelled with RhoDex effectively accepted and accumulated Sterolight probe from U-2 OS donor cells (outlined) within 1–24 h (Fig. 7A). In 24 h, the majority of Sterolight signal sequestrated into acceptor NPC1-RhoDex cells. In the reverse situation, when NPC1 mutated cells were used as donor cells, transfer of the Sterolight signal to RhoDex-labelled U-2OS cells (outlined) was noticeably low even after 24-h co-cultivation (Fig. 7B). To confirm this observation, Sterolight transfer from normal unmutated (HDFa) fibroblasts and two different clones carrying NPC1 mutations (GM18436 and GM03123) to acceptor DF-1-mCherry was performed in parallel (Supplementary Fig. S10). Similarly, in DF-1-mCherry cells, transferred Sterolight signal was weak when mutated NPC1 cells were used as donors. A stronger signal was apparent after 24 h if normal HDFa cells were used as donor. Thus, cells with NPC1 mutation can take up and accumulate Sterolight effectively, but they release this probe into the environment moderately.

## Discussion

The intended purpose of this study was to investigate how the Sterolight sterol probe enters cells, travels inside, gets out of cells, and how reliably it can monitor cellular cholesterol. The pharmacological inhibition by pitstop 2 (a declared clathrin inhibitor) indicated that Sterolight entry may be mediated by the clathrin pathway (Fig. 1), but the results with chlorpromazine (CPZ) and siRNA targeting clathrin heavy chain did not confirm this notion (Supplementary Fig. S2, Fig. 2). In contrast, both inhibitors, consistently inhibited clathrin dependent endocytosis (CDE) of transferrin receptor, which is known as the CDE cargo protein (Supplementary Fig. S2). In this context, it should be noted that some recent work calls into question the specificity of pitstop 2 inhibitor and shows that it also interferes with other molecular targets and inhibits clathrin-independent endocytosis (CIE)^49,50^. Therefore, the results obtained with pitstop 2 must be taken with caution when it comes to distinguishing CDE from CIE^30^. The alternative explanation that Sterolight uptake could be mediated by clathrin-independent endocytosis appears to be supported by several observations. (1) Nystatin, a sterol-binding agent disassembling caveolae and cholesterol in the membrane^51^, inhibits Sterolight uptake (Fig. 1B). (2) Knockdown of endogenous caveolin-1 and dynamin-2 expression, as well as dynamin inhibition by dynasore, resulted in the suppression of Sterolight uptake (Figs. 1A and 2A). (3) In addition, cholesterol under certain conditions may be internalized from the plasma membrane via the caveolar pathway^52,53^. Caveolae-mediated endocytosis is associated with the function of two major and interacting proteins caveolin-1 and dynamin-2^54^. Moreover, CAV1/caveolae and membrane cholesterol are directly and functionally linked to each other^55,56^, thus the membrane cholesterol level appears regulated by CAV1-mediated caveolar pathways^57^. However, the explanation that Sterolight uptake proceeds through caveolae-mediated endocytosis is not consistent with the fact that this event depends on energy^58,59^, whereas in our experiments we observed Sterolight uptake even in cells deprived of energy (Fig. 3A). One possibility is that the energy poisons did not achieve complete elimination of metabolic energy, which is necessary to block the Sterolight uptake at physiological temperature. This does not appear to be the case, however, as the uptake of a transferrin conjugate, known to be dependent on the clathrin pathway, was significantly suppressed in the same cells (Fig. 3A). On the other hand, Sterolight transport to the plasma membrane and cell interior was dramatically reduced at 4 °C (Fig. 3B), in line with previous reports describing temperature dependence of cholesterol transport^60–62^. Nevertheless, this inhibition is more likely related to a drop of membrane fluidity at low temperature^61^. Interestingly, energy-independent cellular influx in energy-depleted cells has been also described for another cholesterol reporter molecule, dansyl-cholestanol^63^. However, dehydroergosterol (DHE) influx was only partly affected by energy poisoning^16^, so there is no full equivalent to our Sterolight probe.

Importantly, there is some evidence in the literature for different energy requirements for cellular uptake of various substances. For example, metabolic inhibitors did not prevent small particles, in contrast to large particles, from entering peritoneal macrophages^64^. Similarly, polystyrene nanoparticles are able to cross the cell membrane through passive, ATP-independent translocation^65^. Further, energy-independent transport mechanisms were reported in the SMase pathway^66^, and in the induction of tubular membrane invaginations by the Gb3 (glycolipid)-binding B-subunit of bacterial Shiga toxin^67,68^. In addition, two metabolic energy-independent pathways were suggested for cell penetrating peptides: (i) direct translocation of the plasma membrane, and (ii) the induction of endocytic-like membrane invaginations (“physical endocytosis” or “self-induced endocytosis”)^69^. Moreover, endocytic internalization events can be mediated by protein-induced rearrangements of a lipid bilayer as a driving force of membrane deformation^70^. One can speculate that the amphiphilic character of the Sterolight molecule and its strong affinity to cholesterol^45^ can facilitate direct translocation from the plasma membrane into the cell interior via non-vesicular transport. However, how would a direct translocation of Sterolight relate to the results of knockdown experiments confirming the involvement of caveolin-1 and dynamin-2? There is a possibility that the probe internalizes into cells via protein carriers. Our results suggest that caveolin-1 could play such a role in Sterolight probe transport. Caveolin-1, a scaffolding protein, has been implicated in numerous cellular functions, including cholesterol export, endocytosis, and the regulation and organization of cell signalling molecules^9,57,59^. Recent works show that caveolins can operate independently of caveolae. These non-caveolar pools of CAV are present in various intracellular membranes including ER, mitochondria, Golgi, endosomes, lysosomes and lipid droplets^59,71,72^. There is an assumption that outside caveolae are caveolins organized as oligomers or scaffolds that generate lipid-ordered domains with the capacity to transport and distribute these lipids to different cellular destinations. They determine the fate of intracellular cholesterol with respect to different trafficking, signalling and metabolic pathways as well as sustain membrane nanodomains in different membranes to remotely regulate protein–protein and protein–lipid interactions and signalling^71,73^. Further, caveolin was shown to move between the plasma membrane and ER, bind cholesterol and preferentially incorporate into liposomes containing cholesterol^74^, indicating its possible shuttling role in cholesterol traffic^55^. Importantly, cholesterol can move directly from the plasma membrane to the ER^75^ and we observed the same movement for Sterolight probe (Supplementary Fig. S5B). Thus, the cholesterol-binding capacity of CAV1 in combination with complex intracellular trafficking, indicate that non-caveolar CAV1 may play a shuttling role in Sterolight trafficking as well. Accordingly, we observed that CAV1 knockdown resulted in the suppression of Sterolight signal in the affected cells (Fig. 2). However, Sterolight uptake was also slightly reduced in cells with knockdown of dynamin 2 and even more in the concurrent absence of clathrin (Fig. 2A). Although CAV1 levels remained in these cells unaffected (Supplementary Fig. S11A), immunostaining revealed that its localization changed. It appeared in clusters in the perinuclear region and these clusters were even more pronounced in the double knockdown (Supplementary Fig. S11B). What is the significance of CAV1 clustering is so far unclear. It is possible that this could change the implied transport role of caveolin, its interaction with other proteins, or cause another effect.

Another explanation, which does not exclude the above, is that the Sterolight uptake includes several mechanisms operating at the same time and this complicates their detection and clear interpretation. The combined action of these pathways makes overall understanding of sterol transport difficult. In addition, we cannot exclude that intense efflux of Sterolight in the form of vesicles may not allow its re-entry into cells by the process described for LDL-cholesterol^76^.

Interestingly, the behaviour, localization and trafficking of Sterolight probe seem to closely correspond to “active cholesterol”, defined as the fraction of cholesterol that exceeds the threshold of complexing capacity of the polar bilayer lipids. It exhibits elevated chemical activity and moves rapidly to intracellular membranes via diverse transport proteins^27,77^. Similarly, Sterolight probe appears in ER and mitochondria shortly after addition to cells, where it might stimulate, like “active cholesterol”, resident effectors to restore the plasma membrane sterol to its resting level (Supplementary Fig. S5). This is indicated by the fact that Sterolight disappears from the ER and mitochondria within 1–2 h and starts to accumulate in lysosomes and later in lipid droplets (Supplementary Fig. S5, S6). At the same time, its efflux occurs to eliminate sterol excess (Fig. 5). For lysosomal translocation, a functional tubulin network is needed, since nocodazole, a disruptor of microtubule polymerization, can prevent it (Fig. 4, Supplementary Fig. S8). In nocodazole treated cell, Sterolight is retained in the ER and mitochondria, without the marked re-localization into lysosomes that is visible in control cells (Fig. 4). This is similar to the results described for caveolin, where nocodazole caused its accumulation in the ER/Golgi intermediate compartment (ERGIC), but subsequent movement to the Golgi was not observed^78^. Thus, this work points to a multi-step process where one of the steps requires microtubules to proceed. If we apply this to Sterolight transport, then this microtubule-dependent step is likely a transport from ER to lysosomes.

Recently, a novel alkyne cholesterol probe^36^ was shown to label ER and mitochondria compartments. However, such localization has not been found in other cholesterol probes such as filipin, DHE and BODIPY-cholesterol^16^. It is possible, that the sub-optimal properties of probes might prevent detection of lower levels of sterol in specific cellular membranes^16^ or that not all cholesterol membrane pools are accessible for particular probes^44^. Similarly, temporary localization in mitochondria and final in lipid droplets was reported for dansyl-cholestanol^79^. Targeting to mitochondria was observed for both 22- and 25-NBD-cholesterol in CHO cells^31^**.** It is obvious that the structure and spectral properties of the probes strongly affects their applicability and some are preferable for certain cholesterol demonstrations than others^80^**.**

The results showing Sterolight efflux appear convincing. Generally, cholesterol efflux plays an important role in maintaining cellular homeostasis. Apolipoprotein A-1 is known as cholesterol acceptor^81^ and accordingly in its presence we observed an intensive Sterolight efflux manifested by the disappearance of Sterolight intracellular fluorescence after 4 h of incubation (Fig. 5). Serum albumin acting as a shuttle to enhance cholesterol efflux from cells^82^ also dramatically reduced Sterolight intracellular signal (Fig. 5). Similar efflux in the presence of BSA and ApoA-I was also described for BODIPY-cholesterol^35^. However, in cells lacking a functional cholesterol transporter Niemann–Pick C1 (NPC1) protein, Sterolight efflux was significantly decreased (Fig. 5B) due to reported defect of cholesterol release to ApoA-I in these cells^83,84^. In addition, Sterolight efflux did not proceed in the presence of energy poisons (Fig. 5C), indicating an active process requiring energy^28,81^. When energy-depleted cells were supplied with glucose, Sterolight efflux was restored (Fig. 5C), just as it was described for DHE efflux^16^. The suppression of Sterolight efflux in the presence of verapamil, the inhibitor of ATP-binding cassette (ABC) transporters, confirms participation of these transporters in Sterolight efflux (Fig. 5D). It is possible that the efflux of Sterolight to ApoA-I is also promoted by ABCA1 and ABCG1 membrane lipid translocases as described for cholesterol^28^.

Other significant results on Sterolight movement were delivered from studies tracking the distribution between different populations of living cells. When U-2 OS cells separately labelled with RhoDex and Sterolight were co-cultured together, a very significant transfer of Sterolight to RhoDex-marked cells was detected within 0.5 to 1 h (Fig. 6). Transfer of Sterolight was faster and more efficient relatively to TF-Chol (Bodipy-Cholesterol), even at 20-fold lower concentration (Supplementary Fig. S12). Inhibitors of actin polymerization (cytochalasin D and latrunculin), but only slightly an inhibitor of microtubule polymerization (nocodazole), interfere with the efficacy of Sterolight transfer. This indicates that tunnelling nanotubes may be relevant for this process (Supplementary Fig. S13). It has previously been shown that actin plays an important role in nanotube formation^85–87^. Therefore, we hypothesized that actin-driven protrusions from donor cells to nearby acceptor cells can facilitate transfer of Sterolight.

Very fast Sterolight transfer was also observed between genetically distinct U-2 OS (Sterolight-labelled) and DF-1 (mCherry-labelled) cells (Supplementary Fig. S9). The efficacy of Sterolight transfer between wild type cells and NPC1-mutant cells was dependent on which cell type was used as a donor or acceptor (Fig. 7). In line with the fact that cholesterol efflux is inhibited in NPC 1 mutated cells, the Sterolight probe was transferred from donor to recipient to a lesser extent if NPC1 was used as a donor (Fig. 7B). When NPC1 cells were used as acceptors, they effectively took up the label, even within 1 h of co-cultivation (Fig. 7A). Sterolight signal intensity in NPC1-acceptor cells increased markedly at the expense of U-2 OS-donor cells and significant sequestration was observed within 24 h (Fig. 7A). When NPC1 mutated cells were labelled with Sterolight and used as donors, probe transfer into RhoDex-labelled acceptor cells was visibly lower (Fig. 7B, C and Supplementary Fig. S10). These results are in line with the fact that cholesterol efflux is inhibited in NPC1 mutated cells (Fig. 5B). On the other hand, a visible amount of Sterolight still appeared in acceptor cells. This indicates that Sterolight transfer from NPC1 mutated cells could be mediated via exosomal release, as described for cholesterol^88^.

**Figure 8:**
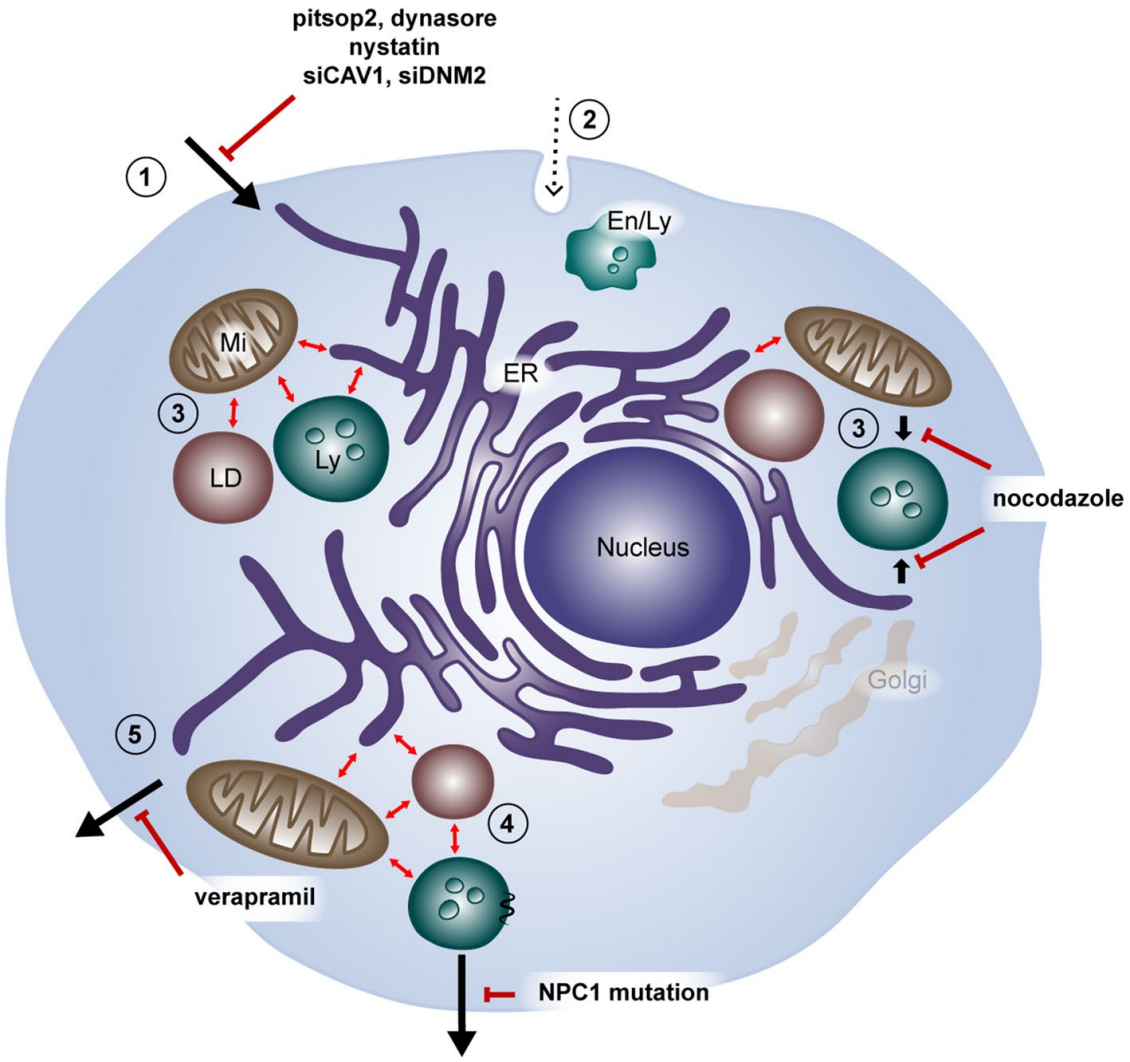
Trafficking itinerary of Sterolight probe. (**1**) Energy independent uptake likely occurs via passive diffusion, penetration or protein carrier-mediated diffusion. It is inhibited by pitstop 2, dynasore, and nystatin inhibitors or by siRNAs-induced knockdown of caveolin-1 and dynamin-2 expression. (**2**) Endocytosis-mediated uptake seems unlikely (dash line). (**3**) Intracellular distribution of the probe occurs very quickly and is likely facilitated by membrane contact sites (MCS) between distinct organelles (shown with red double arrows). Probe temporarily occurs in ER and mitochondria (Mi) and within 1–6 h proceeds to lysosomes (Ly). Transition to lysosomes is blocked by microtubule depolymerizing drug nocodazole. Distribution and transfer inhibition of probe from ER to lysosomes resembles closely intracellular traffic of non-caveolar caveolins. (**4**) Prolonged incubation results in the accumulation of probe excess in lipid droplets (LD). (**5**) Probe efflux is energy dependent and requires functional NPC1 cholesterol transporter. Cells with mutated NPC1 are not able to release accumulated probe from lysosomes as effectively as non-mutated cells. The involvement of ABC transporters in probe efflux is demonstrated by its inhibition with verapamil.

The lessons learned on Sterolight probe uptake, transport and efflux are summarised in Fig. 8. Our results show that uptake takes place through energy-independent process(es), where more mechanisms seem to be involved. One possibility is that Sterolight enters cells by direct translocation and its intracellular transport is associated with caveolin, and/or other potential carriers and further facilitated by membrane contact sites (MCSs) between distinct organelles. This process appears to be multi-step and one of its transfer phases from ER to lysosomes requires the participation of functional microtubules. The Sterolight trafficking strongly corresponds to the concept of previously described "active cholesterol". Due to efficient Sterolight entry into cells, the restoration of the disturbed sterol homeostasis is achieved by storing its excess in lipid droplets and by strong extracellular efflux. This efflux is likely vesicular and energy-dependent. The Sterolight probe is efficiently transmitted between different cell populations, including genetically distinct ones, but is reduced from cells with a mutated NPC1 gene. Although the exact mechanism of how the Sterolight probe enters cells is not fully understood, it does exhibit superior properties, due its high solubility in aqueous solutions, fluorescent intensity, effectiveness at low concentrations and its fast cellular uptake. In summary, Sterolight is suitable for a number of studies, including live imaging, efficient labelling of various cellular compartments, and the tracking and visualization of intracellular and intercellular sterol transfer. Thus, the Sterolight probe expands the currently available repertoire of sterol probes.

## Materials and methods

### Media and supplements

RMPI (21870076), FluoroBrite DMEM (A1896701), foetal bovine serum (FBS) (10270), Opti-MEM reduced serum media (3198504) and supplements including NEAA (nonessential amino acids) (11140-035) were from Thermo Fisher Scientific, lipoprotein deficient serum (LPDS) (S5394) and EMEM medium (M2279) from Merck.

### Inhibitors

Nocodazole (M1404), chlorpromazine hydrochloride (C8138), dynasore (dynamin inhibitor I) (C8138), cytochalasin D (C2618) and latrunculin A (428021) were from Merck. Pitstop 2 (ab120687) and negative control–pitstop 2 (ab120688) were from Abcam, and verapamil was from SiR-tubulin kit (SC002) provided by Spirochrome.

### Antibodies

Anti-clathrin heavy chain antibody (ab21679), anti-dynamin 2 antibody (ab3457) were obtained from Abcam. Caveolin-1 polyclonal antibody (PA5-17447), Alexa Fluor 488 goat anti-rabbit IgG (A11008) and horseradish peroxidase-conjugated donkey anti-rabbit IgG antibody (31458) were from Thermo Fisher Scientific.

### Probes and trackers

Sterolight, whose synthesis and characterization was described previously^45^, was provided by Michal Jurášek (ICT, Prague). Alexa Fluor 568-transferrin (23365), ER Tracker Blue-White DPX (E12353), MitoTracker Deep Red FM (M22426), LysoTracker Red DND-99 (L7528), Rhodamine B-labelled dextran (RhoDex; 10 kD) (D1824), Hoechst 33324 (62249), Draq5 (62251) were from Thermo Fisher Scientific. Lipi-Red (LD03) from Dojindo Molecular Technologies. Filipin III (F4767) was from Merck.

### siRNAs

ON-TARGETplus SMARTpool human DNM2 (L-004007–00-0005), ON-TARGETplus SMARTpool human CLTC (L-004001-01-0005), ON-Targetplus SMARTpool human CAV1 (003467-00-0005), ON-TARGETplus Non-targeting control pool (D-001810–10-05) all from Dharmacon.

### Other chemicals

Fatty free BSA (A7030), ApoA-1 (A0722), methyl-β-cyclodextrin (MβCD) (C4555), 2-deoxy-D-glucose (D8375) were purchased from Merck, Lipofectamine 2000 (11668027), Western lighting chemiluminescence reagent (32132) and ProLong Diamond antifade mountant (34076) from Thermo Fisher Scientific.

### Cell cultures

U-2 OS cells (obtained from ATCC) were cultivated in RPMI 1640 medium supplemented with 10% FBS, sodium pyruvate, 2 mM glutamine, penicillin, streptomycin, 20 mM HEPES, and glucose (4 mg/mL) (Merck). Prior to probe application, the medium was exchanged for a FluoroBrite containing 5% LPDS. Chicken DF1 cells consistently expressing mCherry were obtained from David Přikryl (IMG ASCR, Prague). The NPC1 mutant human fibroblasts obtained from the NIGMS Human Genetic Cell Repository at the Coriell Institute for Medical Research (repository clone number GM03123 and GM18436) were maintained in EMEM medium supplemented with NEAA and 15% FCS. Human dermal fibroblasts (HDFa) from Thermo Fisher Scientific were grown in DMED medium supplemented with 10% FBS.

### Labelling of cells with fluorescent probe Sterolight and organelle markers

Solution of Sterolight probe was prepared in DMSO and applied to cells in cultivation medium supplemented with 5% LPDS at 200 nM final concentration. In specified cases, Sterolight probe (1 µg/mL) was complexed with methyl-β-cyclodextrin (MβCD) at a molar ratio of 1:10 (probe:cyclodextrin), sonicated two times for 3 min and centrifuged for 5 min. Cell cultures were pulsed with the complex for 1 min at room temperature, washed out and chased for 30 min.

For co-localization studies, cells were incubated firstly with probe Sterolight and subsequently loaded with organelle markers LysoTracker Red DND-99 (80 nM), MitoTracker Deep Red FM (100 nM), ER-Tracker Blue-White DPX (250 nM) for 30 min at 37 °C in complete medium, or Lipi-Red (1 µM) in serum free medium. Rhodamine B-labelled dextran (0.5–1 mg/mL) was supplied overnight in normal growth medium.

### Fluorescence microscopy

Cells grown on coverslips in 35-mm Petri dishes were incubated with the Sterolight probe for indicated time in FluoroBrite DMEM medium without phenol red. Subsequently cells were washed and observed alive using a fluorescence microscope DM IRB (Leica) with filter cube I3 (excitation filter BP 450–490 nm and long pass filter LP 515 nm for emission) for green fluorescence. Filter cube N2.1 (excitation filter BP 515–560 nm and long pass filter LP 590 nm for emission) for red fluorescence, and filter cube A (excitation filter BP 340–380 nm and long pass filter LP 425 nm for emission) for blue fluorescence. The fluorescence images were acquired by a DFC 480 camera using a 63 × oil immersion objective as reported before^45^. All results shown are representative of at least 3 separate experiments.

### Inhibitor application

For cytoskeletal disruption, U-2 OS cells were labelled with Sterolight probe complexed with MβCD for 2 min, washed, and chased for indicated time (0.5–4 h) at 37 °C in the presence or absence of 50 µM nocodazole. Endocytosis inhibitors were administered as follows, cells were pre-incubated in the presence of 10 µM pitstop 2 or pitstop 1 for 15 min, or 10 µM dynasore for 1 h, pulsed with Sterolight probe complexed to MβCD and chased for 30 min in the presence of inhibitors in FluoBrite medium with 5% LPDS. For the last 5 min of incubation, Hoechst was included to stain the nuclei.

### Energy depletion

U-2 OS cells were preincubated for 30 min in Medium 1 containing 150 mM NaCl, 5 mM KCl, 1 mM CaCl_2_, 1 mM MgCl_2_, 5 mM glucose and 20 mM HEPES (pH7.4) or Medium 2, which was identical to medium 1, except that it contained no glucose but the energy poisons 5 mM sodium azide and 50 mM 2-deoxyglucose^89^. Next, cells were pulsed with Sterolight probe for 1 min, washed in corresponding media and chased in the presence of Alexa Fluor 568-transferrin (5 µg/mL). Hoechst 33324 (final concentration 1 µM) was added into medium for last 5 min of incubation to stain the cell nuclei.

### Temperature effect

Cells were preincubated for 15 min at 4 °C in FluoroBrite DMEM medium with 5% LPDS and labelled at the same temperature by adding 0.5 µM Sterolight probe for 30 min. Hoechst (1 µM) was included for last 5 min of incubation to stain the nuclei.

### siRNA transfections

Transfections were performed according to the Thermo Fisher Scientific siRNA transfection protocol﻿﻿.﻿﻿﻿﻿ Briefly, U-2 OS cells were plated in 2 mL of growth medium without antibiotics on 30 mm dishes to reach next day approximately 30–50% density. Prior to transfection, the cells were washed in serum-free and antibiotic-free Opti-MEM and incubated for 1 h in the same medium. For small interfering RNAs (siRNA) experiments were used 25 nM human CAV1 (caveolin-1), CLTC (clathrin heavy chain), DNM2 (dynamin-2) or control ON-TARGET pools of siRNAs. Lipofectamine and each siRNA were diluted in 250 µL Opti-MEM and combined together for 20 min to form oligomer-Lipofectamine complexes. Then, the complexes were added directly to cells in 1 mL of serum-free and antibiotic-free Opti-MEM. After 6 to 8 h incubation at 37 °C in a CO_2_ incubator, cells were supplemented with an additional 1 mL of cultivation medium containing serum at final concentration 10%. In two days, cells from each transfected variant were split into three dishes and 24 h later subjected to analyses. Two dishes of each variant were used for verification of knockdown by immunofluorescence and Western blot analysis. The third dish was examined for effect of specific knockdowns on uptake of Sterolight probe. For this purpose, cells plated on coverslips were washed with serum free medium, pulsed for 1 min with complex of Sterolight probe (0.5 µg) with MβCD, washed again and chased for 30 min in medium containing 5% LPDS. For the last 5 min of incubation, Hoechst (1 µM) was added to stain nuclei.

### Western immunoblot analysis

Cellular extracts were prepared from siRNA transfected cells in RIPA buffer. Proteins were separated concurrently with a protein ladder (Thermo Scientific Page Ruler) ranging from 10 to 170 kDa) in 8–12% SDS-PAGE gels followed by electrophoretic transfer to Amersham-Hybond ECL nitrocellulose membrane. Full-length membranes were blocked for 1 h in 5% non-fat dry milk and incubated with relevant primary antibodies diluted in TBS-T (1:1000) containing 5% BSA. Subsequently, the blots were rinsed three times with washing buffer (TBS-T) and incubated with horseradish peroxidase-conjugated donkey anti-rabbit IgG secondary antibody (1:10,000) for 1 h. After five rinses of the membranes in washing buffer, protein expression was visualized by Western lighting chemiluminescence reagent or by SuperSignal West Dura reagent. Equal protein loading and transfer was verified by Ponceau-S staining of the membrane and actin re-probing. If, for spatial reasons, we show only a cropped part of the blot with a corresponding bend of protein, full-length membranes with marked margins are presented in the Supplementary Information in several exposures.

### Immunofluorescence

Cells plated in glass coverslips coated with poly-L-lysine were after washing fixed with 3.5% paraformaldehyde for 10 min and washed in PBS. After fixation, cells for clathrin and dynamin-2 immunofluorescence were incubated in PBS-T (0.1% Tween in PBS) containing 1% BSA and 0.3 M glycine for 1 h to permeabilize the cells and block non-specific protein–protein interactions. Cells were labelled with anti-clathrin and anti-dynamin 2 antibody diluted in PBS-T (1:200) with 1% BSA for 1 h at room temperature and then labelled with Alexa Fluor 488 goat anti-rabbit secondary antibody diluted 1:500 in PBS-T. Hoechst 33342 was added to label the cell nuclei for the last 5 min of incubation. After washes in PBS, coverslips were embedded in ProLong Diamond antifade mountant and epifluorescence examined under a fluorescence microscope. The permeabilization of cells for caveolin-1 immunofluorescence was done in PBS containing 0.1% Triton TX -100 for 15 min and then samples were blocked with 1% BSA for 1 h at room temperature. Afterwards, cells were labelled with caveolin-1 rabbit polyclonal antibody at 1:100 dilution in PBS with 0.1% BSA, incubated at 4 °C overnight and then labelled with secondary antibody as described for clathrin and dynamin.

### Lipid droplets isolation

U-2 OS cells (3 × 10^7^) were incubated with Sterolight for 48 h, washed with PBS and scraped from dishes with rubber policeman to a centrifuge tube. After additional washing and homogenization, the supernatant containing a floating fat layer was collected, and lipid droplets were isolated by density gradient centrifugation^90^. The isolated lipid droplet fraction was extracted and analyzed by LC–MS.

### Lipid extraction for LC–MS analysis

Lipids were extracted from cell lysates based on a previously described method^91^. The cell lysates (1 ml) were extracted by adding 3.75 ml of chloroform–methanol (1:2 v/v), vortexed and centrifuged at 2600 rpm for 5 min to pellet insoluble material. The supernatant was transferred to a glass tube and 1.25 ml each of chloroform and PBS were added. Samples were vortexed and centrifuged again. The lower chloroform layer was evaporated to dryness under a weak stream of argon and the solid residue was dissolved in an acetonitrile–water (3:7 v/v) mixture (0.5 ml), filtered using a syringe filter (0.2 µm, PTFE), and transferred to the autosampler glass vial.

### LC–MS analysis

The lipid extracts were analysed using a UHPLC-MS system (Waters). The instrument comprises of the binary LC pump (Acquity UPLC I-Class), Sample Manager and Autosampler (Flow Through Needle ACQUITY UPLC I-Class), Column manager (CM-A), eλPhotodiode Array Detector (PDA), and single-quadrupole mass detector (SQ2 Detector).

The extracted lipid mixture was separated by loading an aliquot (2 µl) onto a heated (50 °C) C18 reverse-phase column (ACQUITY UPLC BEH C18 130 Å, 1.7 µm, 2.1 mm × 50 mm) using a gradient elution. The 5 min gradient was formed by mixing solvent A (water with 0.05% formic acid) and B (acetonitrile with 0.035% formic acid) starting at 97% of A, moving to 5% of A at 3.75 min, and back to 97% of A at gradient end (5 min).

The analytes were detected by a combination of optical (photodiode array—PDA) and molecular mass (electrospray ionization—ESI) detection. Continuous scanning of *m/z* 100–1000 Da in both positive and negative ionization modes provided the total ion current (TIC) chromatograms from which the extracted -ion chromatograms were generated.

First, the analytical standards of Sterolight and its free-hydroxyl form derivative FP-7 were analyzed. Calibration (dilution series measurement) of both analytes was carried out and limit of detection (LOD), limit of quantification (LOQ) and linearity parameters were determined. Sterolight (exact mass 694.43 Da) eluted at a retention time (RT) of 2.71 min and FP-7 (exact mass 652.43 Da) eluted at RT = 2.27 min.

The lipid extracts were analysed using the above-described LC–MS methodology to monitor the ratio of the Sterolight and FP-7 over the course of 48 h in cell lysate, in lipid droplets, and for transesterification products detection.

### Statistical analysis

The microscopic images were analysed by the Fiji software using a custom-made macro. Images were manually segmented to contain one cell per file. Cells were thresholded on the images smoothened by Gaussian blur. Threshold values were determined either by autothreshold by Li and Tam^92^, or set manually to correctly contain the entire cell when autothreshold provided poor results. Intensity of the fluorescence in the thresholded area was subsequently measured in the unblurred images. The mean fluorescence intensity was determined and statistical analysis of 15–40 cells was carried out using one way Anova. Values p < 0.01 were considered significant. Sterolight transfer from donor to acceptor cells was evaluated as percentage of acceptor cells with visible signal.

## Supplementary Information

Supplementary Information.

## Data Availability

All data supporting the findings of the present study are contained in the manuscript or the supplementary file. Additional raw data are available upon request to corresponding author.
