# Supplementary material for: Sterolight as imaging tool to study sterol uptake, trafficking and efflux in living cells
Source: Sci Rep. 2022 Apr 15;12:6264. doi: 10.1038/s41598-022-10134-x (PMC9012876; doi:10.1038/s41598-022-10134-x)
Supplement: Supplementary file 1 — Supplementary Information. [file 41598_2022_10134_MOESM1_ESM.pdf]

SUPPLEMENTARY INFORMATION

Sterolight as imaging tool to study sterol uptake, trafficking, and efflux in living cells

Jarmila Králová[1], Martin Popr[1], Jan Valečka[2], and Petr Bartůněk[1]

[1]CZ-OPENSCREEN, Institute of Molecular Genetics of the Czech Academy of Sciences, Prague, Czech Republic

[2]Light microscopy core facility, Institute of Molecular Genetics of the Czech Academy of Sciences, Prague, Czech Republic

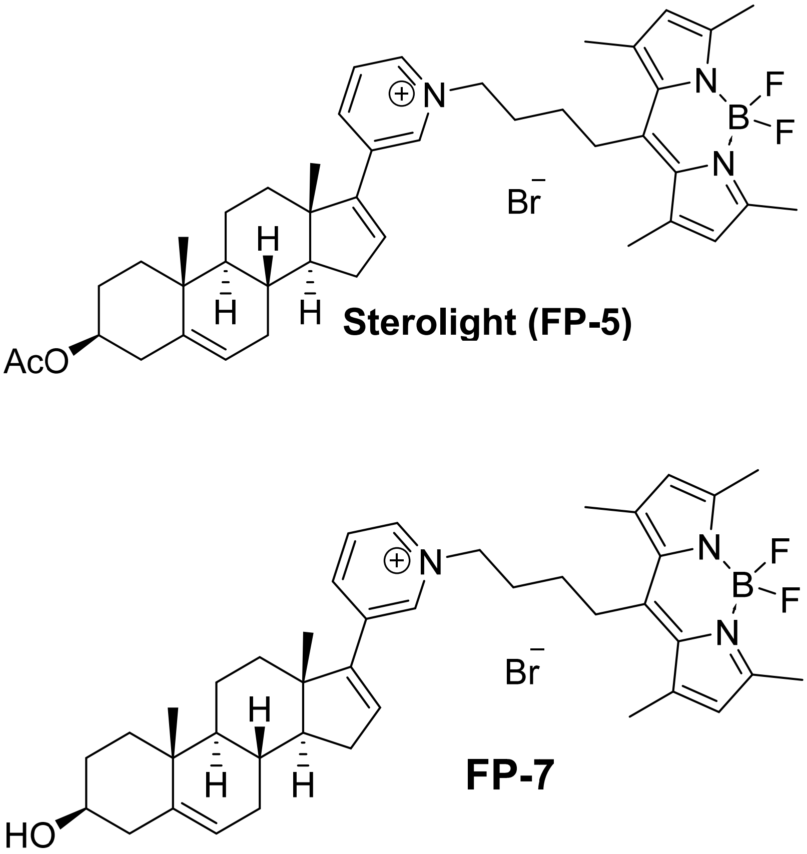

Sterolight (FP-5)

FP-7

Figure S1. Structures of sterol sensing probes Sterolight (acetylated form) and FP-7 (hydroxyl-derivative).

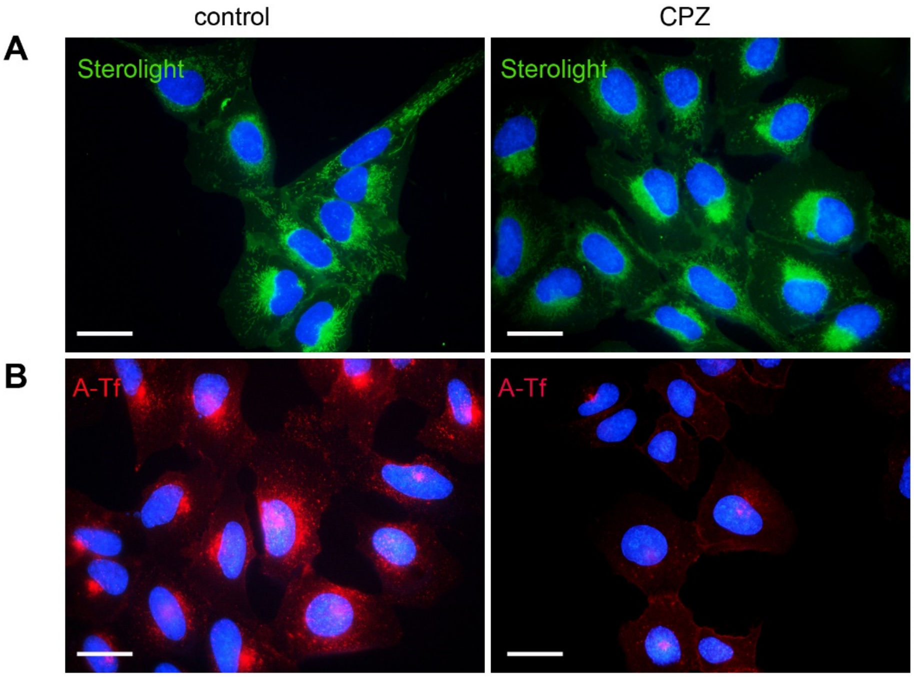

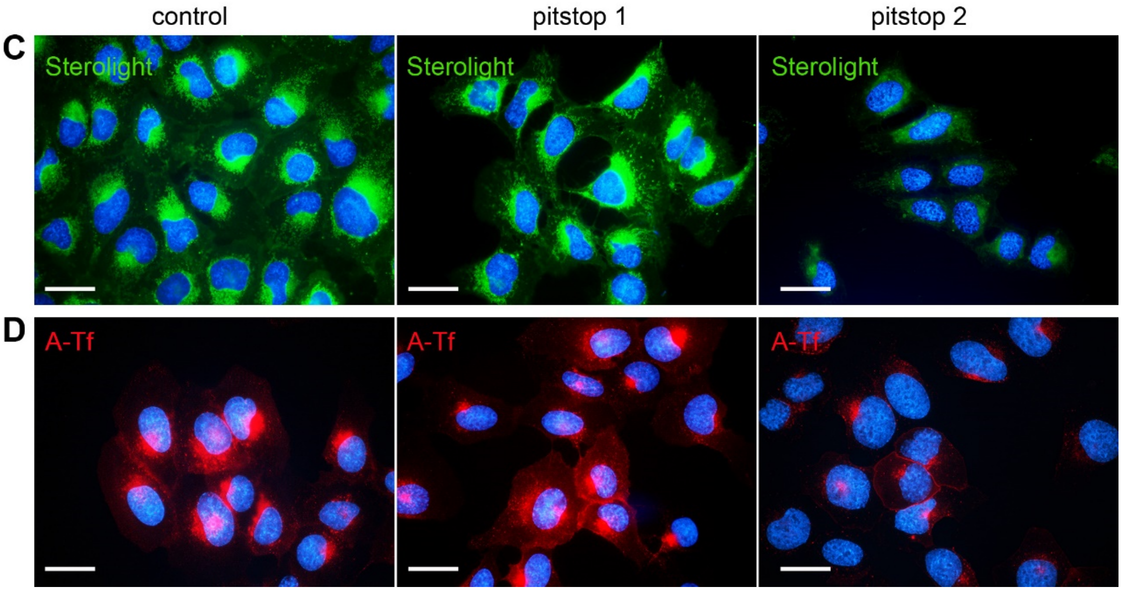

Figure S2. Effect of chlorpromazine and pitstop treatment on Streolight and Alexa 568-transferrin uptake. (A, B) U-2 OS cells were pre-incubated with 10 μM chlorpromazine (CPZ) for 30 min and then either pulsed with Sterolight (A) or incubated in the presence of 20 μg of Alexa 568-transferrin (A-Tf) (B) for additional 30 min. CZP treatment substantially reduced A-Tf, but did not affect Sterolight uptake. (C, D) U-2 OS cells were pre-incubated with 10 μM pitstop 1 and pitstop 2 for 15 min in serum free medium. Subsequently, cells were pulsed with Sterolight complexed with MβCD and chased in the presence of inhibitors in FluoroBrite medium with 5% LPDS (C). Cells were after pre-treatment incubated in the presence of Alexa 568-transferrin (A-Tf) and inhibitors for 30 min (D). Pitstop 2 treated cells exhibited markedly reduced Streolight and A-Tf signal in contrast to pitstop 1 treated cells used as negative control. Cell nuclei were stained for the last 5 min of incubation with Hoechst 33342. Scale bar 10 μm.

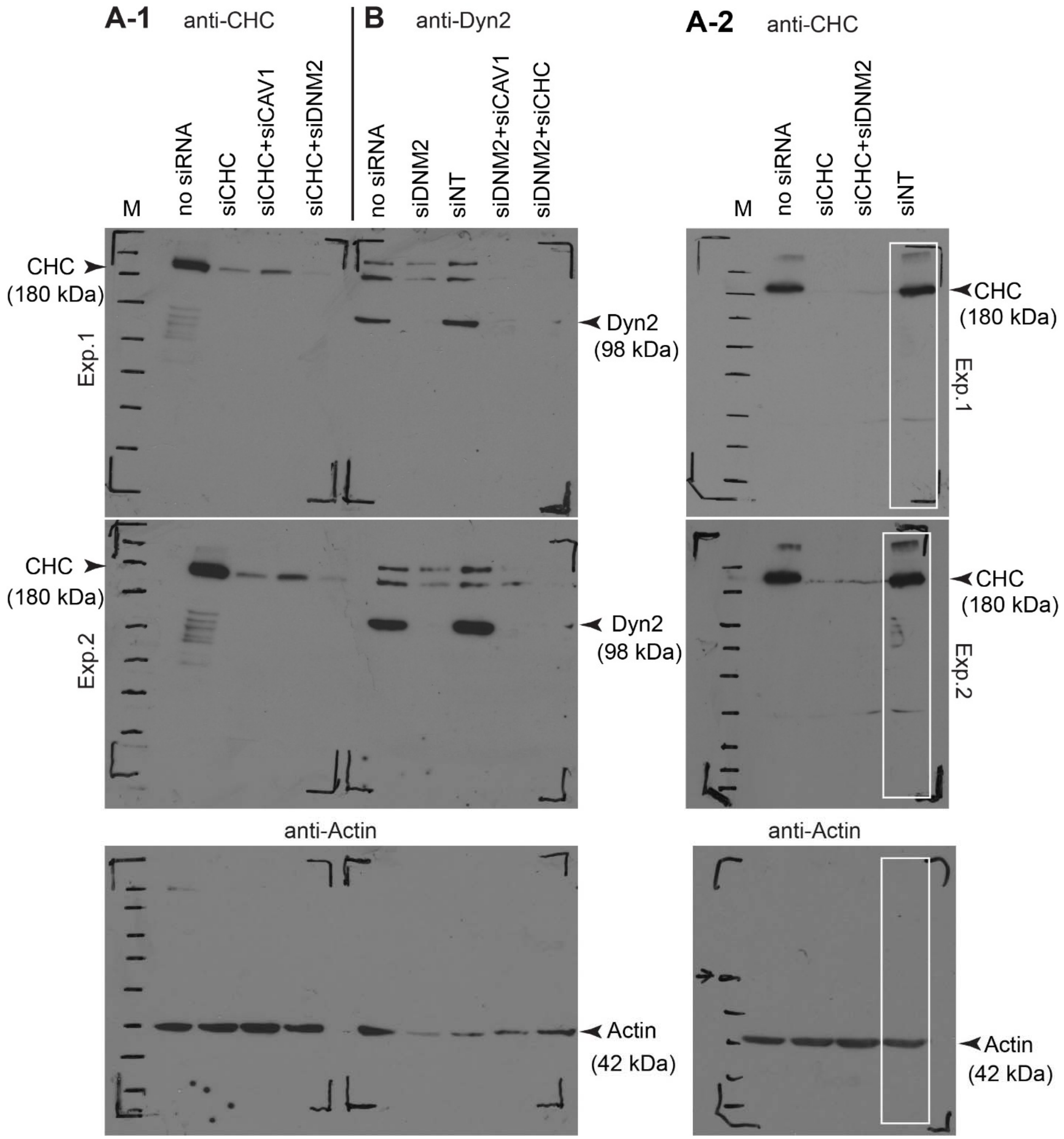
A-1
anti-CHC
B
anti-Dyn2
A-2
anti-CHC
M
no siRNA
siCHC
siCHC+siCAV1
siCHC+siDNM2
no siRNA
siDNM2
siNT
siDNM2+siCAV1
siDNM2+siCHC
M
no siRNA
siCHC
siCHC+siDNM2
siNT
CHC
(180 kDa)
Exp.1
Dyn2
(98 kDa)
CHC
(180 kDa)
Exp.1
CHC
(180 kDa)
Exp.2
Dyn2
(98 kDa)
CHC
(180 kDa)
Exp.2
anti-Actin
Actin
(42 kDa)
anti-Actin
Actin
(42 kDa)

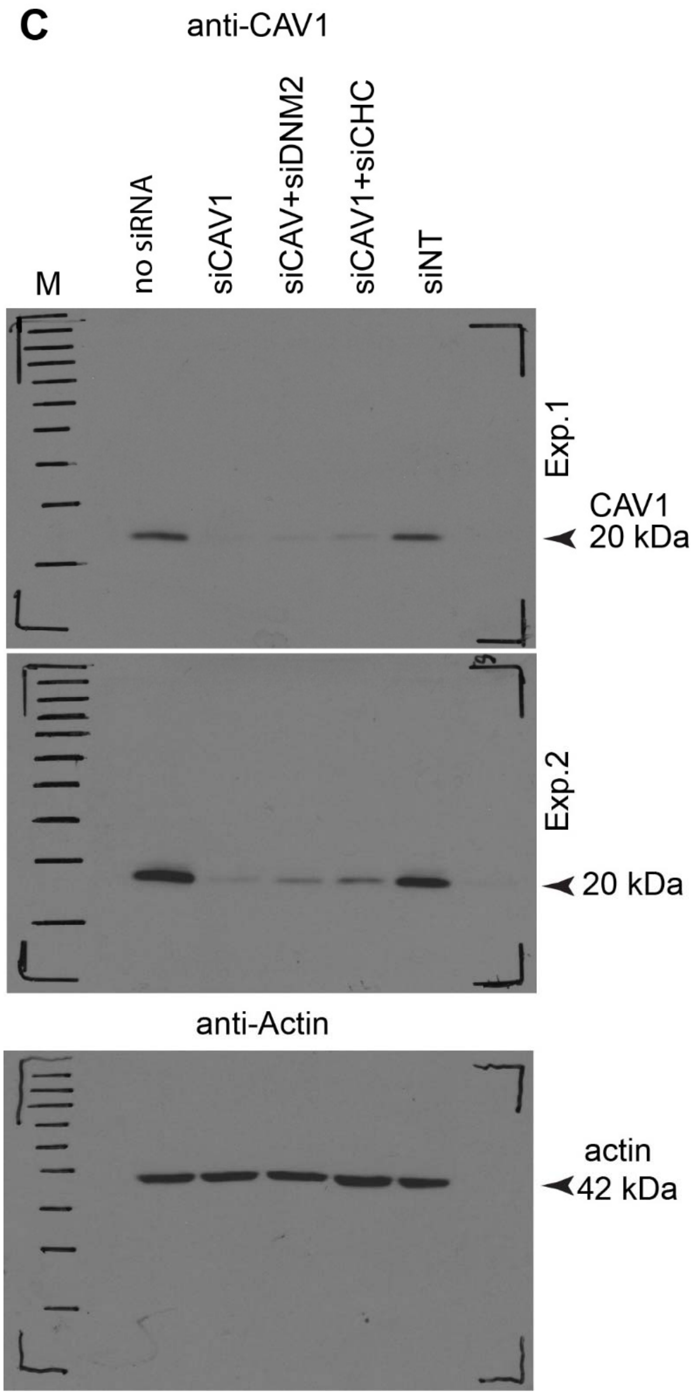

Figure S3. Images of full-length Western blots demonstrating knockdown of caveolin-1, dynamin-2 and clathrin presented in Figure 2 in the article with different exposures. The same blots were stripped off and re-probed with actin. A) Blots detecting CHC: A-1 was combined with part of A-2 (framed band siNT and corresponding actin). B) Blot detecting dymanin 2, re-probed with actin (shown beside). C) Blot detecting CAV1, re-probed with actin (below).

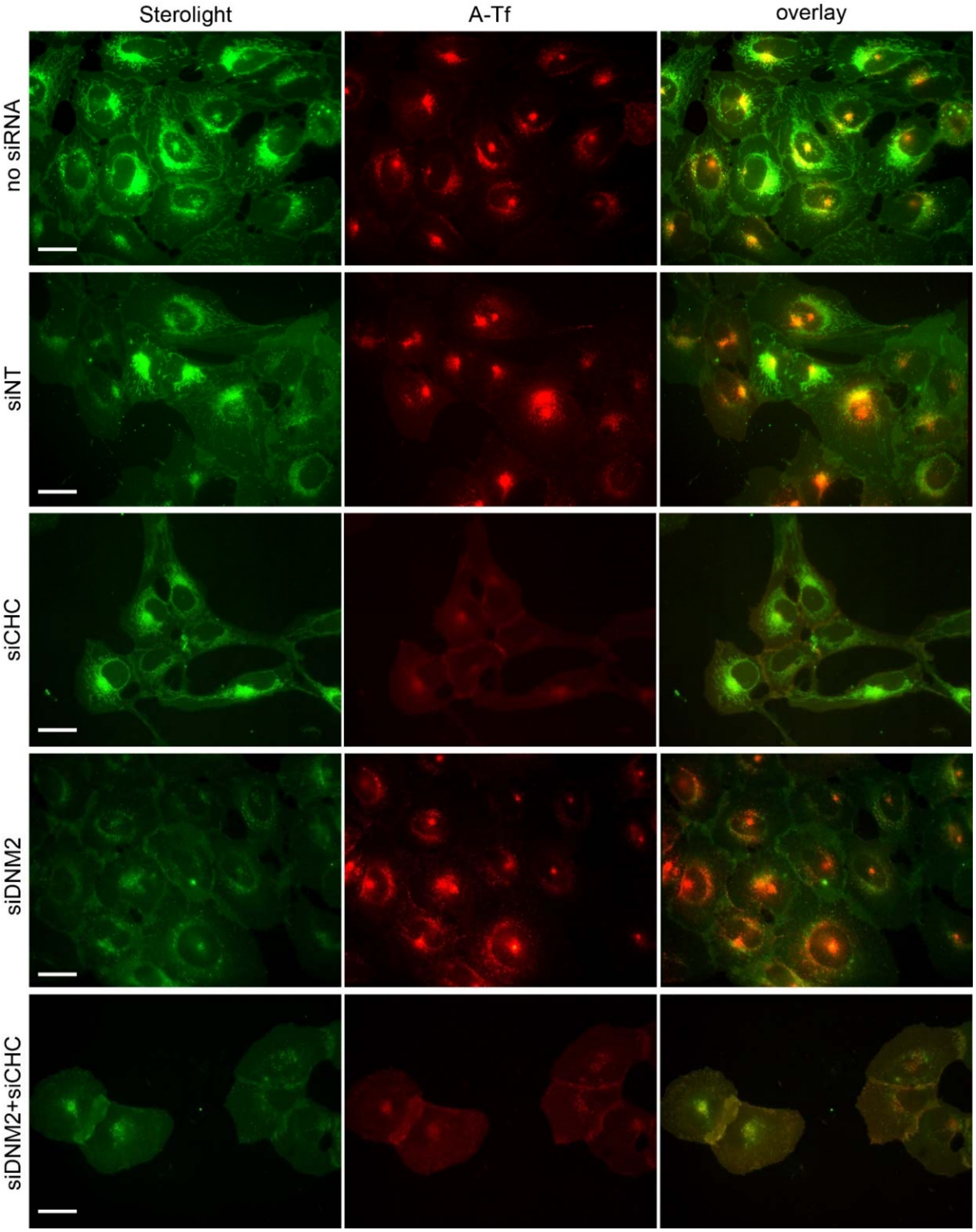

Figure S4. The effect of clathrin and dynamin 2 depletion on the uptake of Streolight and Alexa 568-transferrin. Cells with silenced expression of specified genes were, 96 h after transfection, pulsed with Streolight (1 μg/ml) for 1 min, washed and chased for 1 h in the presence of Alexa 568-transferrin (Tf-A). For comparison, cells subjected to transfection without siRNA (no siRNA) or cells transfected with non-targeting siRNA (siNT) were included as negative controls. Clathrin depletion (siCHC) alone or in combination with DNM2 resulted in a low A-Tf signal, while Sterolight signal in siCHC remained strong as in controls. A decreased level of the Streolight signal occurred only in combination with dynamin, which might be attributable to the effects of siDNM2. Scale bar 10 μm.

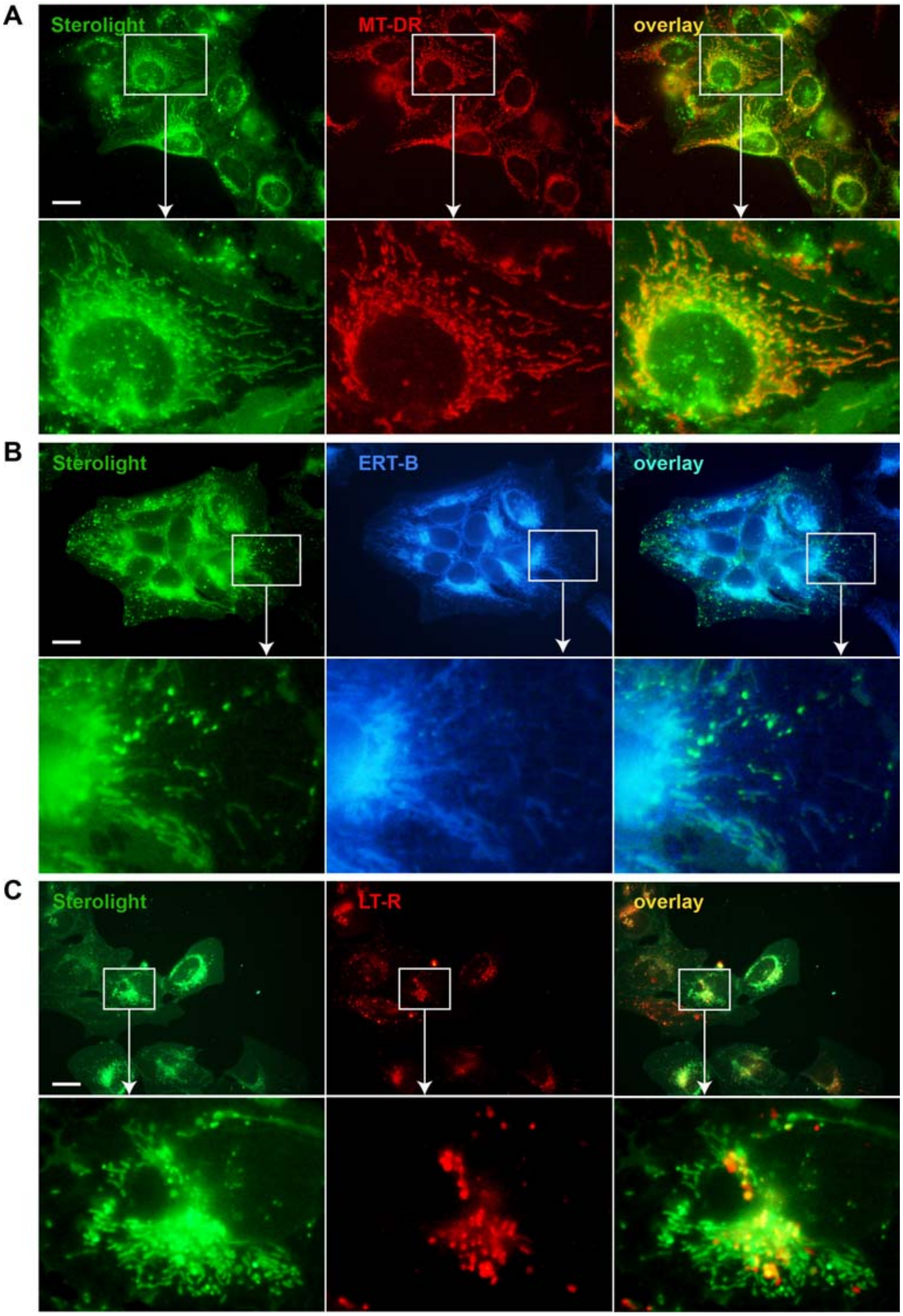

Figure S5. Co-localization of Sterolight with organelle markers in U-2 OS cells. Cells labelled with Sterolight were chased for 0.5 and 1 h in the presence of markers: A) 100 nM MitoTracker Deep Red (MT-DR), B) 250 nM ER-Tracker Blue-White DPX (ERT-B), or C) 80 nM LysoTracker Red DND-99 (LT-R). Enlargements of areas marked with white boxes are shown in the bottom row of each panel. Sterolight signal co-localized temporarily with mitochondria and ER. Subsequently increasing content of Streolight in lysosomes is demonstrated by co-localization with lysosome marker LT-R. Scale bar 10 μm.

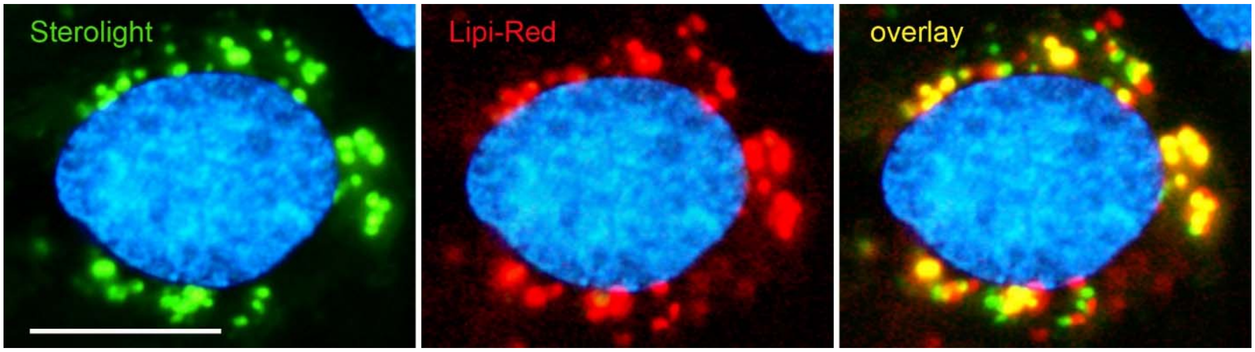

Figure S6. Co-localization of Sterolight with Lipi-Red. U-2 OS cells were labelled for 48 h with 200 nM Sterolight in medium containing 5 % LPDS. Subsequently cells were stained in serum free medium for 30 min with 1 μM Lipi-Red and for the last 5 min with Hoechst 33342 to label nuclei. Co-localization of Sterolight and Lipi-Red in lipid droplets is indicated by yellow colour. Scale bar 10 μm.

A

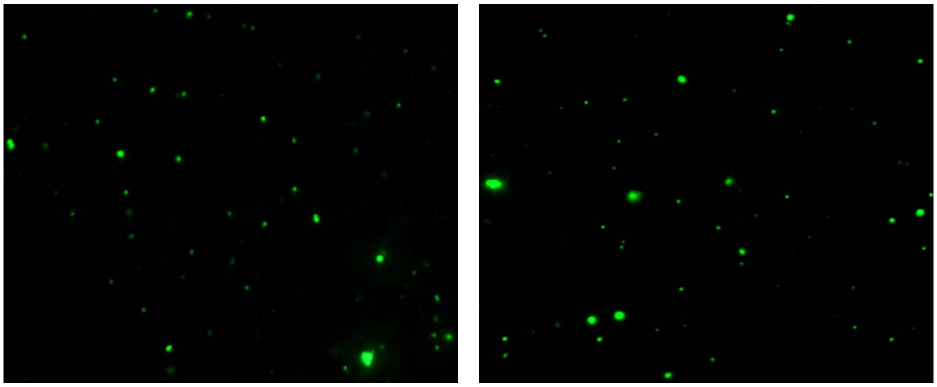

B

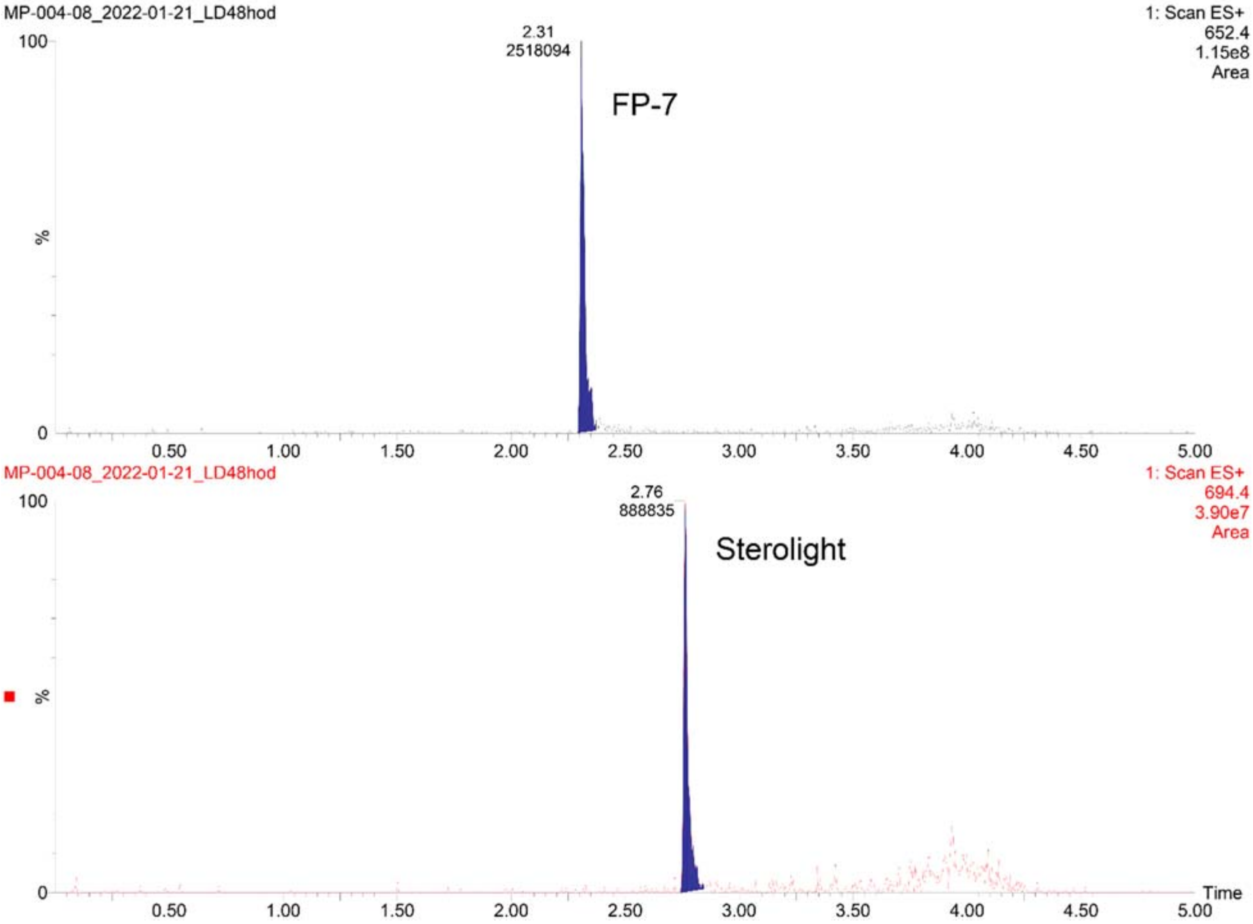

Figure S7. LC-MS monitoring of Sterolight forms in the lipid droplets after 48-hours cell treatment. (A) Fluorescence imaging of isolated lipid droplets fraction. (B) Extracted-ion MS chromatograms detecting both acetylated (Sterolight) and hydroxyl (FP-7) forms (ratio 1:2.7).

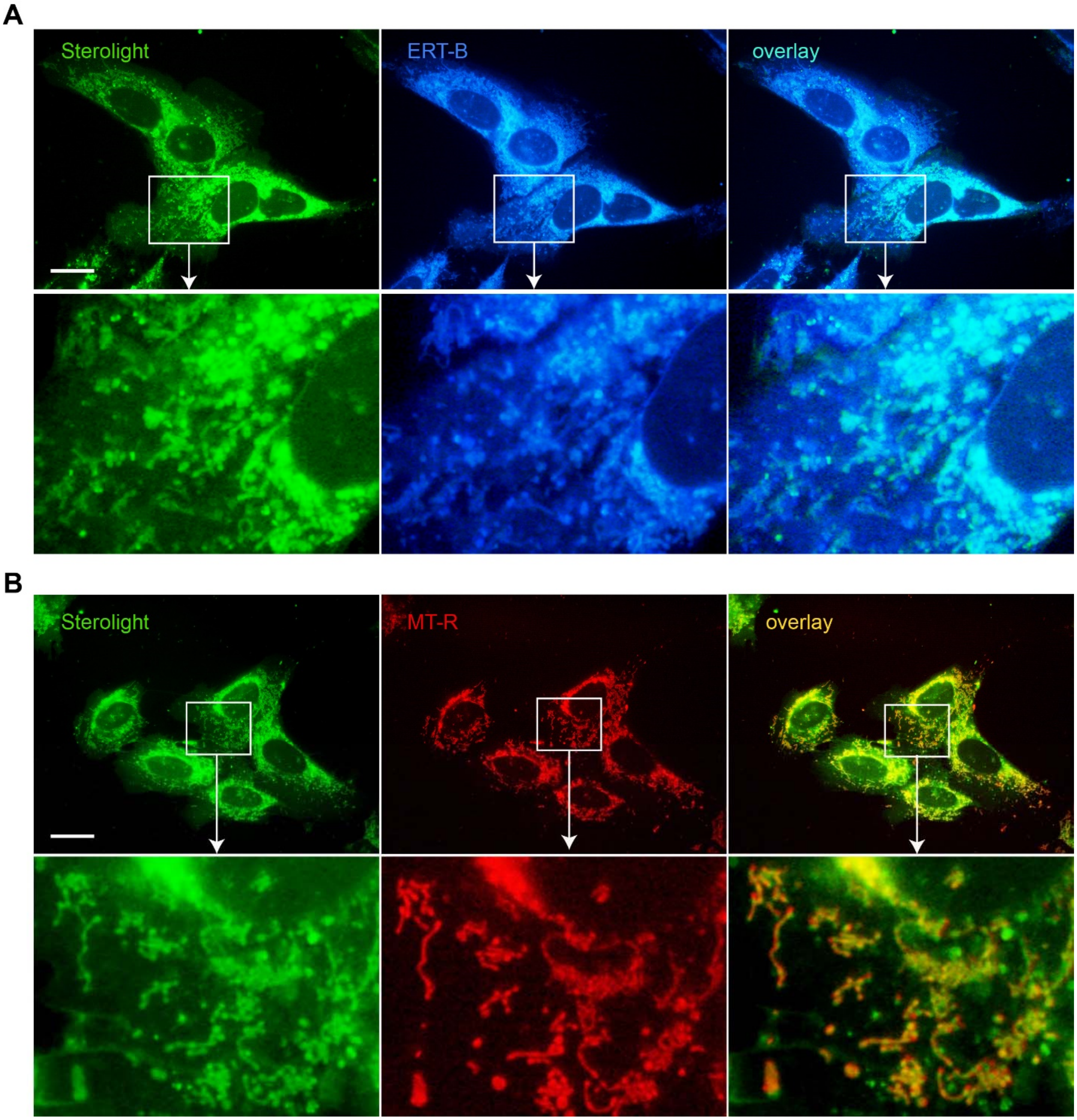

Figure S8. Localization of Sterolight signal in nocodazole treated cells. U-2 OS cells were pulsed with Sterolight (1 µg) complexed to MβCD for 1 min and chased in the presence of 50 µM nocodazole for 1 h. A) The markers ER Tracker Blue (ERT-B) (200 nM) and B) MitoTracker Red (MT-R) (10 nM) were added for the last 30 min of incubation. Enlargements of areas marked by white boxes are shown in the bottom of each panel. Sterolight signal co-localized partly with ER and mitochondria. Scale bar 10 µm.

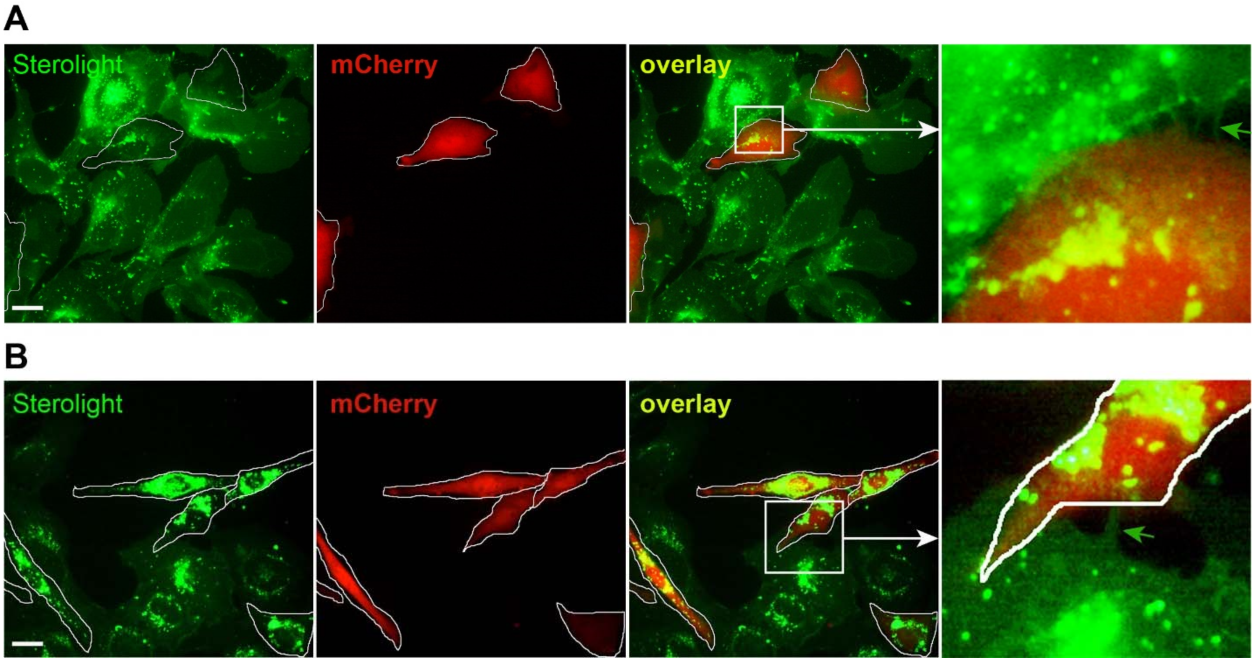

Figure S9. Co-cultivation of Sterolight labelled U-2 OS cells with DF-1 chicken cell line stably expressing mCherry. A) DF1cells expressing mCherry (outlined) were seeded on the plate with Sterolight labelled U-2 OS cells. Mixed population of cells were co-cultivated for 30 min (A), or 24 h (B). The fluorescence of the same field was recorded using different filters for Sterolight (green), mCherry (red). Enlargements of areas marked by white boxes are shown to the far right. Nanotubes between Sterolight and mCherry labelled cells indicating sterol transfer to DF-1 cells are shown by green arrowheads. Scale bar 10 μm.

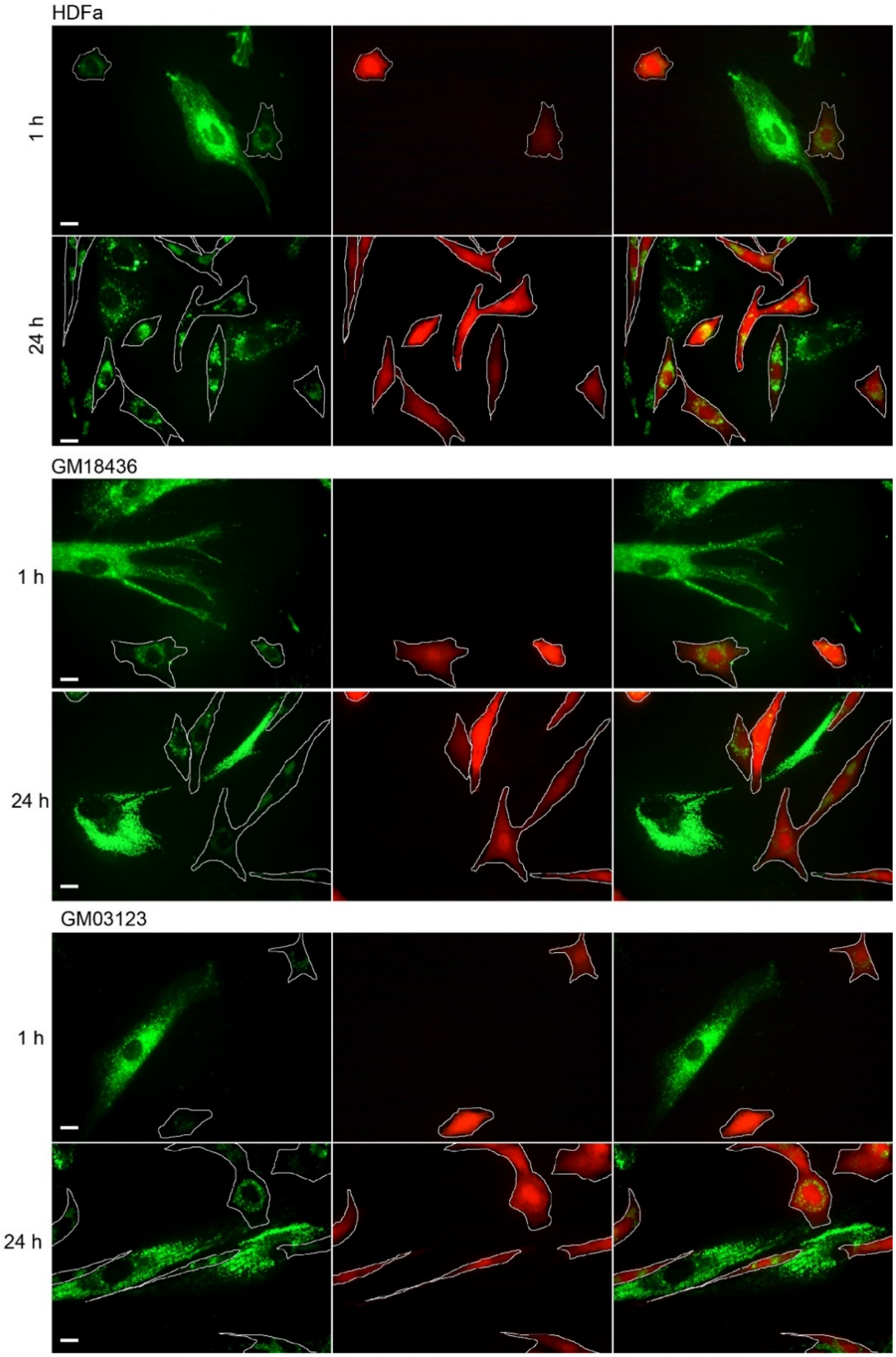

Figure S10. Sterolight transfer between Sterolight-labelled fibroblast human cell lines and chicken DF1-mCherry line. DF1 cells stably expressing mCherry (outlined) were seeded with human dermal fibroblasts HDFa or clones with mutated NPC1 (GM18436 and GM03123) labelled with Sterolight probe. Mixed population of cells were co-cultivated for 1 or 24 h. The fluorescence of the same field was recorded using different filters for Sterolight (green), mCherry (red). Scale bar 10 μm.

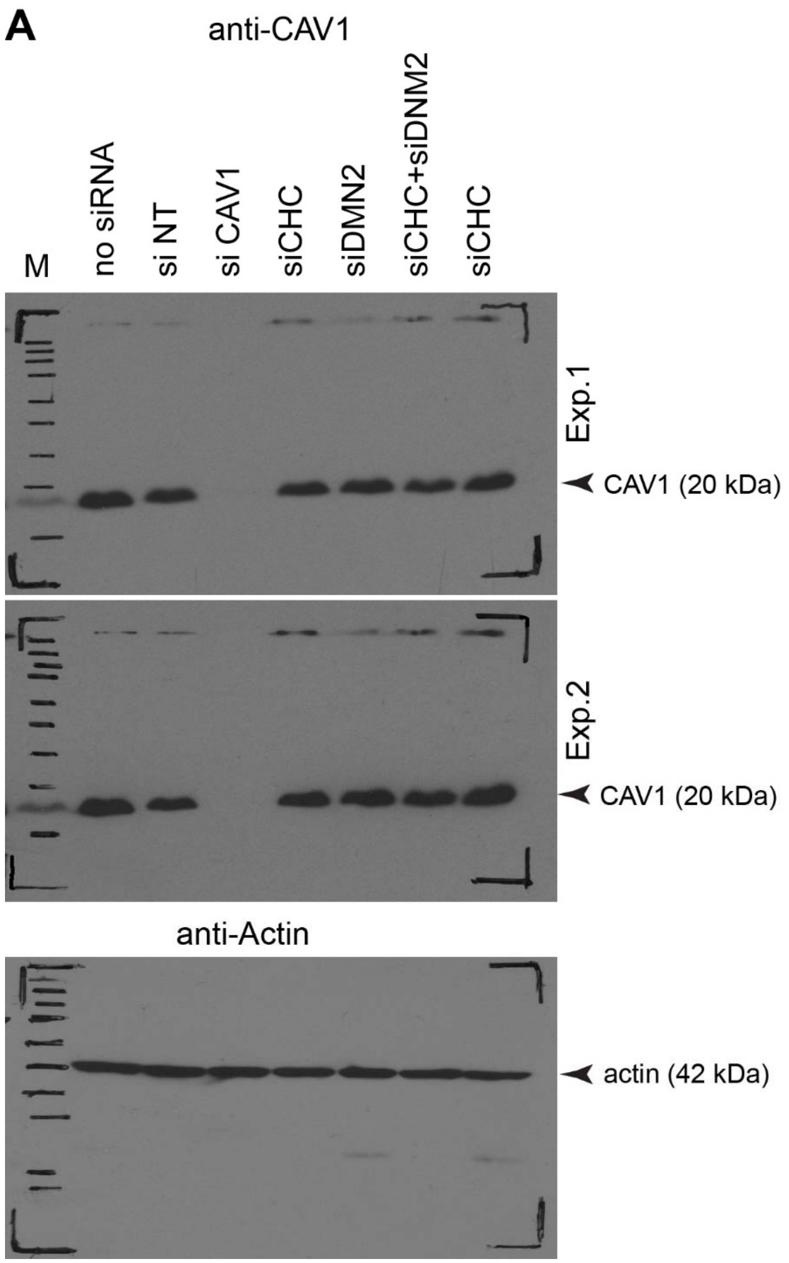
A
anti-CAV1
M
no siRNA
si NT
si CAV1
siCHC
siDMN2
siCHC+siDNM2
siCHC
Exp.1
CAV1 (20 kDa)
Exp.2
CAV1 (20 kDa)
anti-Actin
actin (42 kDa)

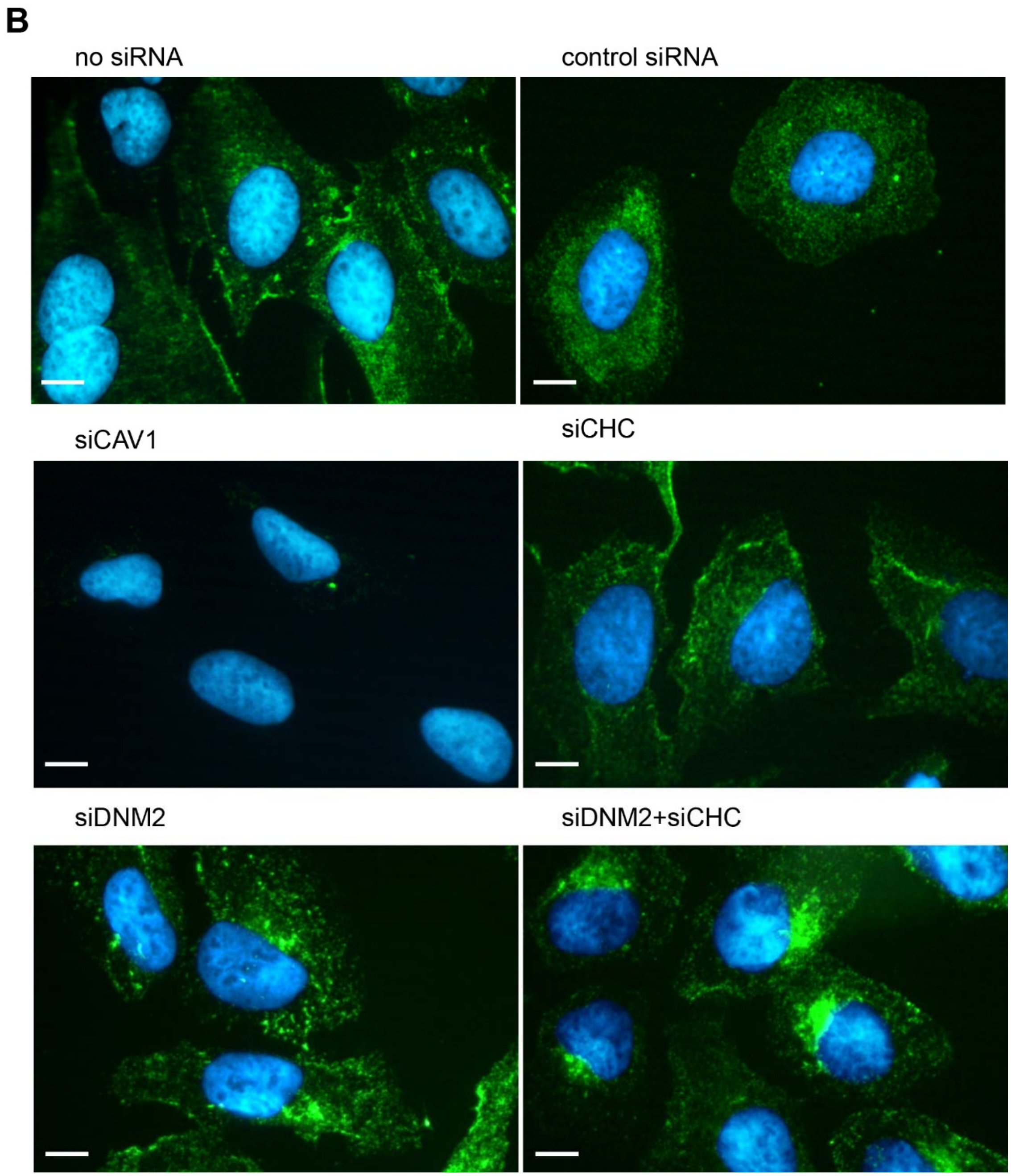

Figure S11. Dynamin-2 knockdown affects localization of CAV-1. U-2 OS cells were transfected with siRNAs targeting clathrin (siCHC), dymamin-2 (siDNM2), caveolin-1 (siCAV1) or combination siDNM2+siCHC. Cells subjected to transfection procedure without siRNA (no siRNA) or cells transfected with non-targeting siRNA (siNT) were included as negative controls. The expression and localization of CAV-1 protein in different transfected variants was assessed 96 h after transfection by Western blot analysis (A) and by immunostaining (B). (A) Images of full-length Western blots (different exposures) display similar expression of CAV-1 protein in controls and the knockdowns of dynamin-2, clathrin, or the combination of these two. The same blots were stripped off and re-probed with actin (below). (B) However, dynamin knockdown correlated with CAV-1 clustering, which was even more pronounced in the perinuclear region when in combination with clathrin knockdown. Cell nuclei were stained with Hoechst for 5 min. Scale bar 10 μm.

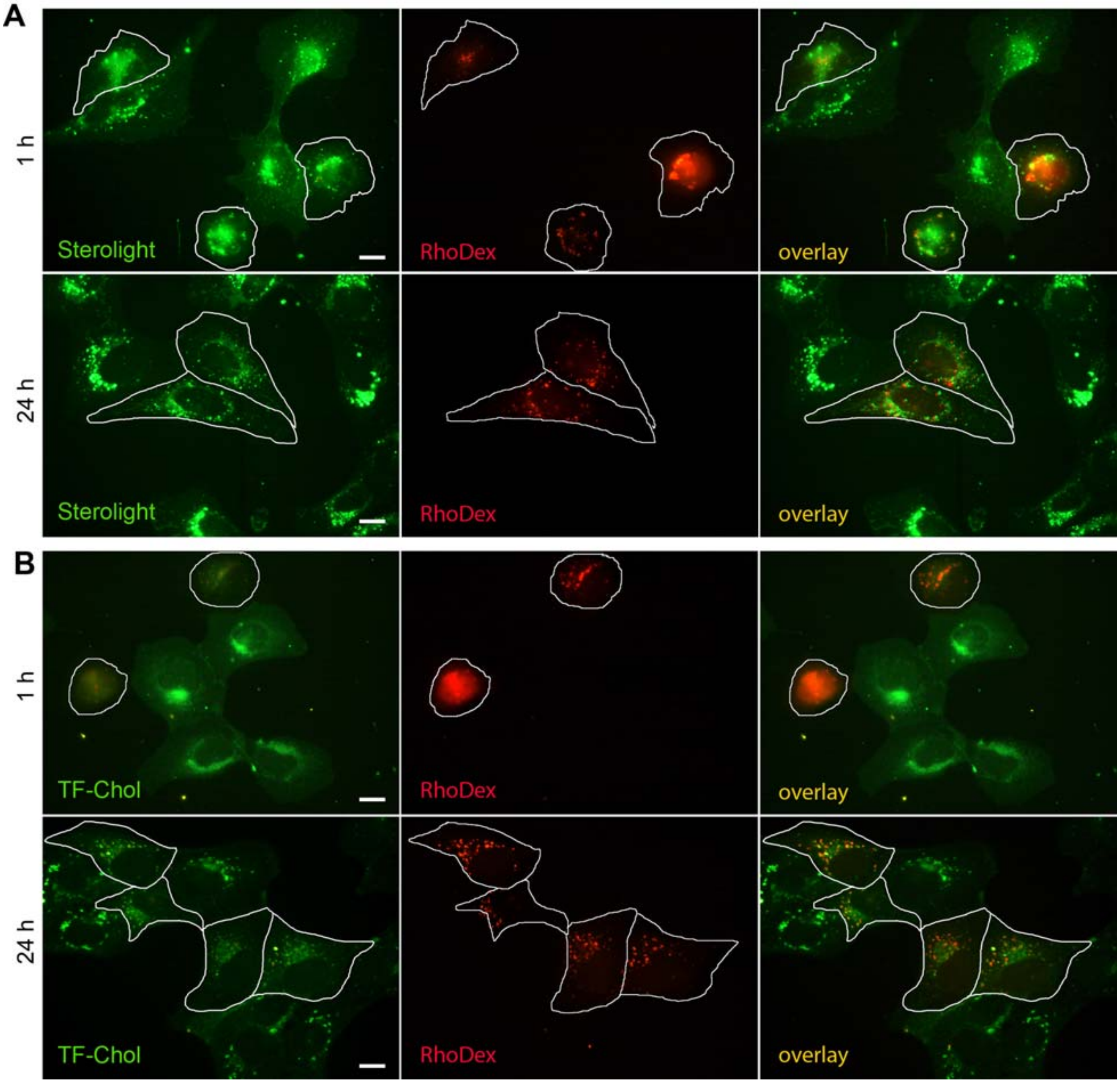

Figure S12. Transfer of Sterolight and TF-Chol probes from donor to acceptor cells. Acceptor U-2 OS cells were labelled overnight with rhodamine-dextran (RhoDex) (0.5 mg/ml), harvested and plated on dishes with U-2 OS cells labelled with Sterolight (1μg) and TF-Chol (20 μg) probes complexed with MβCD (ratio 1:10) *via* pulse for 1 min. RhoDex (outlined) and sterol labelled cells were further co-cultivated in FluoroBrite medium supplemented with 5% LPDS for 1 h or 24 h. The same cells were scanned for green and red fluorescence using identical setting for each probe. Sterolight probe was quickly effluxed from donor cells (green) and taken by RhoDex (red, outlined) acceptor cells within 1 h (A), while TF-Chol probe transfer was slower and less effective (B). Scale bar 10 μm.

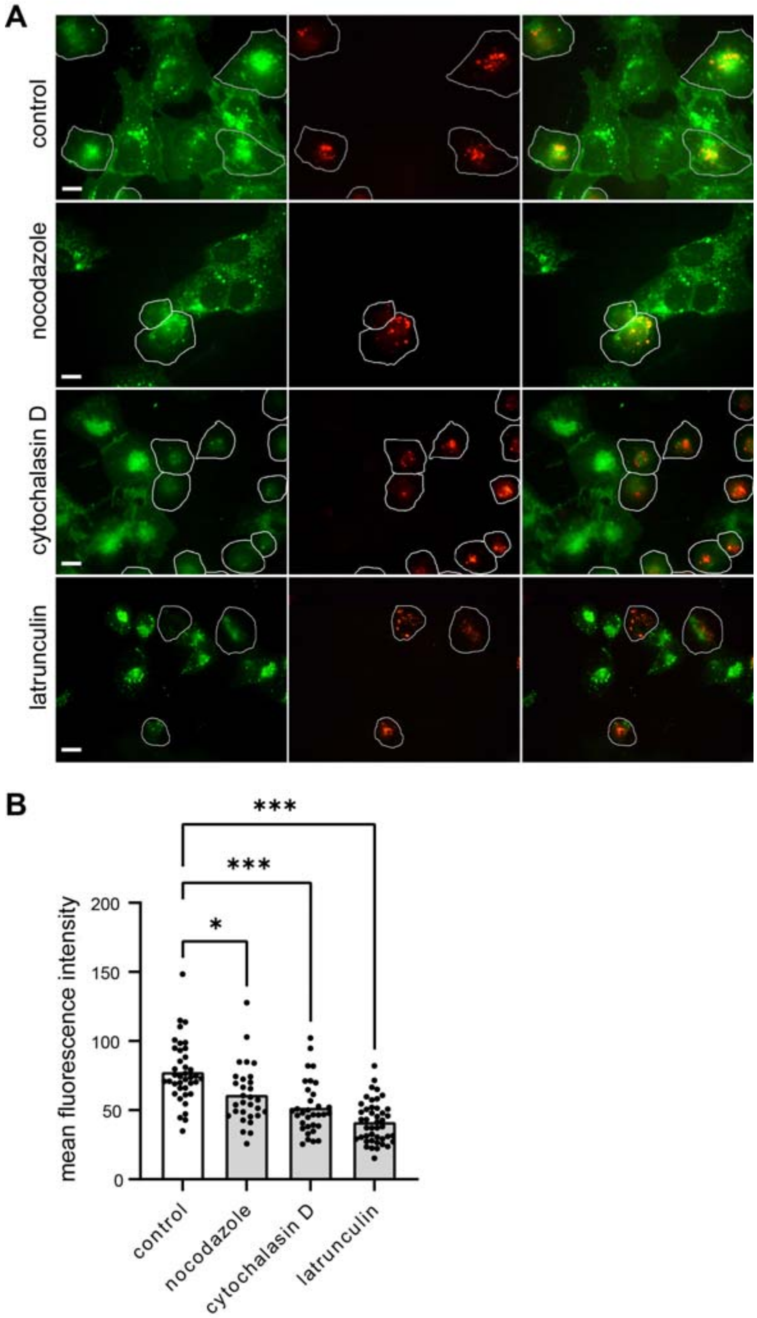

Figure S13. Sterolight transfer to acceptor cells is partly suppressed by actin polymerization inhibitors. The effect of inhibitors of actin polymerization (cytochalasin D, latrunculin), and microtubule polymerization (nocodazole) on Sterolight transfer to rhodamine dextran (RhoDex) labelled U-2 OS cells was assessed. (A) Sterolight pulsed cells were pre-incubated for 30 min with 20 μM nocodazole and then RhoDex labelled cells (outlined) were added and co-cultivated for an additional 30 min before Sterolight transfer examination. Pre-incubation with cytochalasin D (1 μM) and latrunculin (0.5 μM) lasted only 10 minutes, followed by 15 - 20 minutes of co-cultivation with RhoDex-labelled cells. This setup was necessary since longer treatment interfered with cell viability as described previously in the literature [1]. (B) Sterolight transfer to RhoDex-labelled cells was significantly reduced (*$p<0.001$) with actin inhibitors but only slightly with nocodazole (*$p <0.05$).

1. Crespo, A. C. *et al.* Decidual NK Cells Transfer Granulysin to Selectively Kill Bacteria in Trophoblasts. *Cell* **182**, 1125-1139 e1118, doi:10.1016/j.cell.2020.07.019 (2020).
